# DNA vaccines targeting hemagglutinin from 18 subtypes of influenza A virus to antigen-presenting cells confer broad protection

**DOI:** 10.1016/j.omtn.2025.102814

**Published:** 2025-12-26

**Authors:** Ane Marie Anderson, Elias Tjärnhage, Daniëla Maria Hinke, Ranveig Braathen, Gunnveig Grodeland, Bjarne Bogen

**Affiliations:** 1 Institute of Clinical Medicine, University of Oslo, Oslo, Norway; 2 Department of Immunology (IMM), Oslo University Hospital, Oslo, Norway; 3 Center for Pandemics and One-Health Research, SUSTAINIT, Institute of Health and Society, University of Oslo, Oslo, Norway

**Keywords:** MT: Delivery Strategies, vaccine, influenza, broad immunity, antibodies, variable viruses, epitope dilution, bivalency, antigen mixture, APC targeting

## Abstract

Novel vaccines that confer broad protection against influenza A viruses (IAVs) are urgently needed. Hemagglutinin (HA) is the major influenza antigen targeted by protective immune responses. We have here developed a DNA vaccine that simultaneously presents HA from 18 subtypes of IAV to the immune system. The vaccine consists of a DNA plasmid mixture that encodes a variety of dimeric vaccine proteins. Each dimer expresses two different HAs, as well as a targeting moiety directing the vaccine protein to antigen-presenting cells (APCs). When the vaccine proteins were targeted toward chemokine receptors 1, 3, and 5 (CCR1/3/5) on APC by means of macrophage inflammatory protein 1-alpha (MIP1α) (CCL3), vaccinated mice were broadly protected against infection with H1N1, H3N2, H5N1, and H7N1 influenza viruses. Furthermore, antibody-mediated protection against H1N1 was maintained when the H1 antigen was removed from the plasmid mixture, indicating that the diversity of HAs in the mixture promoted formation of antibodies specific for shared, conservative epitopes. The results may guide the development of a broadly protective influenza A vaccine for humans.

## Graphical abstract

**Figure:**
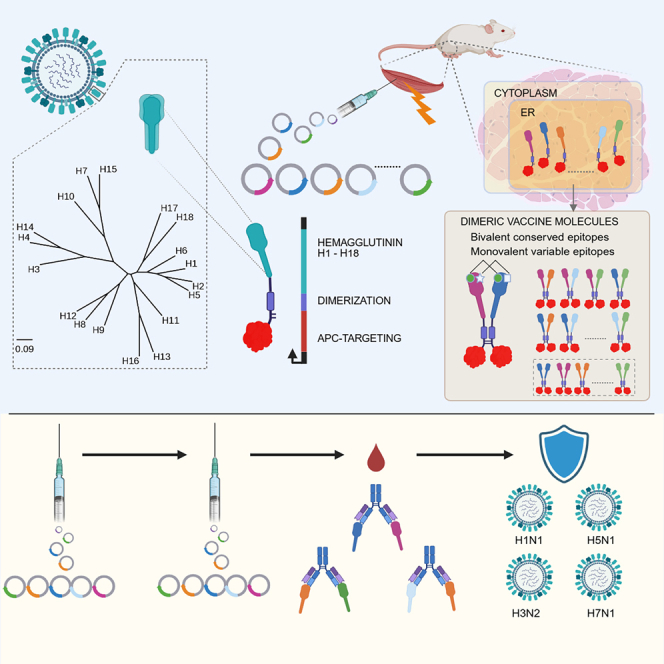

## Teaser

Grødeland, Bogen, and colleagues have mixed hemagglutinin from 18 different subtypes of influenza to dilute strain-specific responses and up-concentrate shared regions. When this mixture presents bivalent hemagglutinins to selected immune cells, immune responses are directed toward shared regions. The result is the development of cross-reactive immune responses that can broadly protect against influenza.

## Introduction

Seasonal influenza viruses cause significant morbidity and mortality, with an estimated global death toll of 290,000–650,000 annually.^1^ Widely circulating human influenza A viruses (IAV) have so far been limited to the H1, H2, and H3 influenza virus subtypes,^2^ but human infections with subtypes from zoonotic reservoirs, such as H5N1 and H7N9, also occur.^3^^,^^4^ Genetic reassortments between human and zoonotic viruses have caused four pandemic influenza outbreaks since 1918.^2^ Furthermore, current vaccines against the annually circulating seasonal influenza variants suffer from frequent mismatches between vaccine strains and circulating strains due to antigenic drift.^5^ Since seasonal influenza vaccines will not protect against the next pandemic, and since we do not know which strain of influenza will cause an outbreak, we cannot prepare pandemic vaccines in advance.

We have previously developed DNA vaccines that rapidly induced protective levels of neutralizing anti-hemagglutinin (HA) antibodies (Abs) after a single vaccination.^6^^,^^7^ These plasmid vaccines encode vaccine proteins that deliver selected antigens directly to antigen-presenting cells (APC), resulting in improved Ab and T cell responses to HA in mice.^6^^,^^7^^,^^8^^,^^9^^,^^10^^,^^11^ Improvement of Ab responses was further enhanced by a dimeric format of vaccine proteins, resulting in bivalent display of antigen. Such antigen bivalency most likely contributes to cross-linking of B cell receptors (BCRs) in APC-B cell synapses and increased B cell responses.^11^^,^^12^ It follows that a combination of two different HA subtypes (e.g., HA_X_ and HA_Y_) in a single dimeric vaccine protein should preferentially promote cross-linking of BCRs specific for conserved epitopes shared between HA_X_ and HA_Y_. This principle, called valency-based immunoselection, has been used to obtain broadly reactive Abs against conserved epitopes shared between different HA subtypes.^13^^,^^14^

When targeting vaccine proteins to receptors on APCs, the choice of receptor and the type of APC can polarize the phenotype of the ensuing immune response.^9^^,^^10^^,^^15^ More specifically, targeting HA to MHC class II (MHCII) molecules on APCs induced T_H_2-skewed responses and IgG1 Abs in mice.^7^ Conversely, targeting HA to chemokine receptors (CCRs) 1/3/5 by use of the chemokine macrophage inflammatory protein 1-alpha (MIP1α) (CCL3) enhanced T_H_1 and CD8 T cell responses, as well as IgG2a Abs.^10^

In our previous studies on DNA-delivered HA mixtures, we targeted vaccines encoding either a mixture of 6^13^ or 16^14^ subtypes of HA toward MHCII molecules on APCs. These vaccines induced protection against two H1N1 viruses that were not present in the vaccine mixtures, but the protection was incomplete for the largest vaccine mixture even after three vaccinations.^13^^,^^14^ We here show that targeting the HA mixtures to APCs by MIP1α significantly improved cross-protection after only two vaccinations. The protection was broad, since vaccinated mice were protected against heterologous H1N1, H5N1, and H7N1 influenza subtypes not included in the vaccine mixtures, as well as homologous H3N2. A comparison of two different ways of dimerizing the vaccine proteins demonstrated a protective favor when randomly dimerizing the HAs for bivalent display, rather than selectively coupling more distant HA antigens, in mixtures containing HA from all 18 influenza subtypes. These results could be readily translatable, since a human equivalent of MIP1α, LD78β,^16^ has given promising results as a targeting unit of papilloma virus cancer vaccines in humans^17^ and advanced cervical cancer.^18^

## Results

### MIP1α-targeted DNA vaccines encoding a single HA subtype induced strain-specific Ab responses

**Figure 1:**
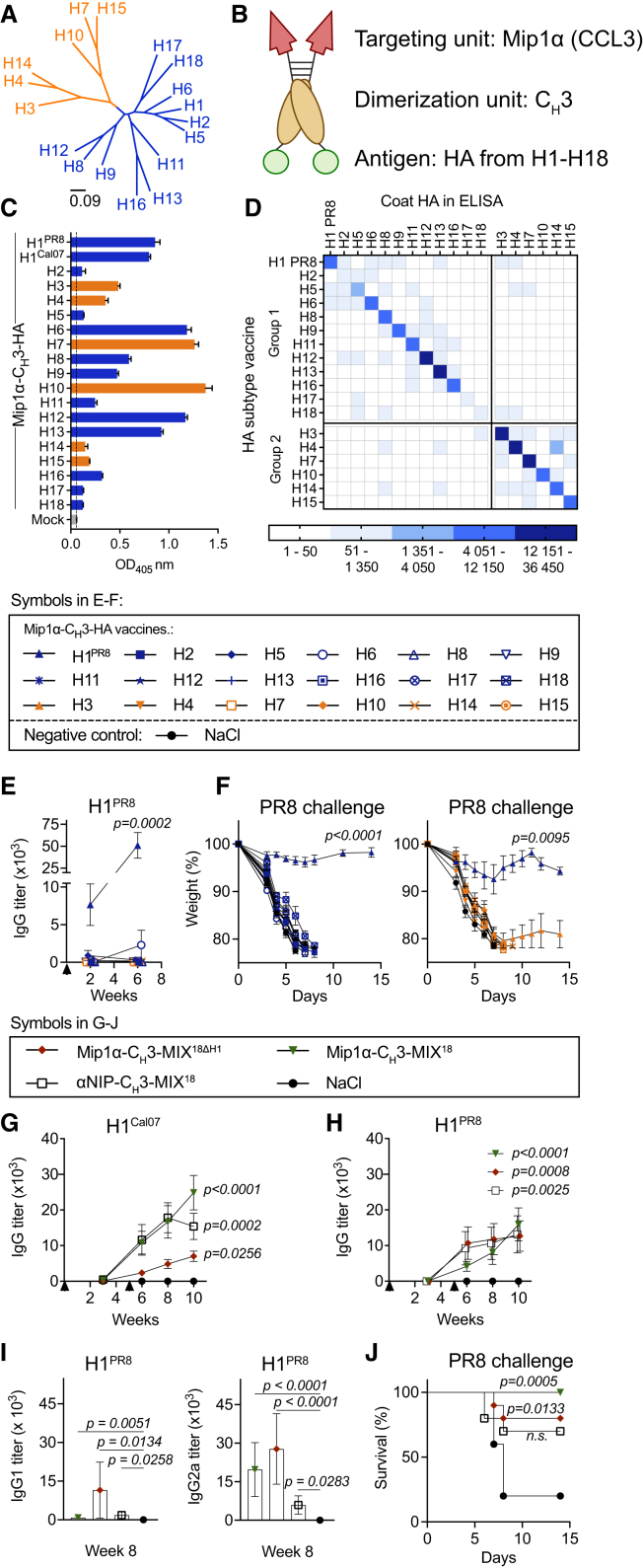
MIP1α-targeted HA vaccines induce cross-reactive antibodies and protection when delivered simultaneously, but not separately (A) Phylogenetic tree showing the classification of group 1 and group 2 HA. The scale bar represents % variation in amino acid sequences (influenza group 1 in blue, group 2 in orange). (B) Schematic of a homodimeric vaccine protein consisting of (i) a targeting unit (the CCR1/3/5 ligand Mip1α [CCL3]), (ii) a dimerization unit (a shortened hinge of exons h1 and h4 and the C_H_3 region from human IgG3), and (iii) an antigenic unit (HA). (C) Secretion of vaccine proteins. HEK293E cells were transiently transfected with 1 μg of each indicated Mip1α-C_H_3-HA plasmid. Secreted vaccine proteins were detected in the supernatant after 72 h by ELISA using an anti-C_H_3 coating antibody and an anti-Mip1α detection antibody (mean ± SEM of technical triplicates). (D–F) BALB/c mice (*n* = 6/group) were immunized once with 20 μg of Mip1α-C_H_3-HA vaccines encoding a single HA subtype (Figure S2A). (D) IgG titers against H1–H18 were measured in sera pooled per group by ELISA at week 6 after immunization (mean, technical triplicates). (E) H1^PR8^-specific IgG was measured in sera at weeks 2 and 6 after vaccination (mean ± SEM, individual mice). (F) Six weeks after vaccination, mice were challenged with influenza PR8 virus (5L×D_50_) and monitored for weight loss (mean ± SEM) in two separate experiments. Mice were euthanized upon reaching 20% weight loss. (G–J) BALB/c mice (*n* = 10/group) were vaccinated twice at weeks 0 and 5 (arrows) with the indicated plasmid mixtures. (G–I) IgG antibodies against H1^Cal07^ (G) and H1^PR8^ (H) were measured in individual mice at weeks 3, 6, 8, and 10, and (I) H1^PR8^-specific IgG1 and IgG2a were measured at week 8 (mean ± SEM). (J) Five weeks after the boost, mice were challenged with PR8 influenza virus (5×LD_50_) and monitored for weight loss. Survival curves represent mice reaching 20% weight loss as the humane endpoint. Statistical significance was calculated using: Kruskal-Wallis with Dunn’s multiple comparisons test in (E) and (G–I), two-way ANOVA with Dunnet’s multiple comparison in (F), and the Gehan-Breslow-Wilcoxon test against NaCl in (J). All groups were compared to NaCl at the final time point in (E), (G), and (H), or on day 8 in (F). Only significant values are shown.

Genes encoding the ectodomain of HA from 18 subtypes of group 1 and 2 IAV (Figure 1A) were inserted into plasmid DNA vectors that, after translation, expressed a fusion protein with (i) an APC-binding targeting unit consisting of the mouse chemokine MIP1α (CCL3) specific for CCR1/3/5, (ii) a dimerization unit consisting of a shortened hinge and C_H_3 from hIgG3, and (iii) an antigenic unit containing one of the 18 HA subtypes. The central location of the C_H_3 dimerization motif in the polypeptide enables formation of X-shaped dimeric vaccine proteins, where the two targeting units point in one direction and the two monomeric HA antigens in the other direction^7^ (Figure 1B). When such a bivalent vaccine protein binds an APC, the two identical HA will point outwards toward the BCR in an APC-B cell synapse.^11^^,^^12^ Representatives of the 18 HA subtypes were selected based on current and past circulation of viruses, isolation from humans, representative phylogenetic traits, and commercial availability of protein reagents (strains in Table S1). The vaccines were named according to their targeting, dimerization, and antigen units, e.g., MIP1α-C_H_3-H1.

To evaluate whether vaccine proteins were produced and secreted from cells, each plasmid was transiently transfected into HEK293E cells, and vaccine proteins secreted into supernatants were measured by sandwich ELISA. All 18 different HA vaccine proteins were detected, but at variable levels (Figure 1C). The vaccines were present in the expected dimeric structures, and the APC-specific targeting units were confirmed to be functional (Figures S1A and S1B). Vaccination in mice raised some Abs against the backbone structures of C_H_3 and MIP1α (Figure S1C), but caused limited inflammation at the site of delivery the day after vaccination (Figures S1D and S1E). The latter point was based on histological evaluations of local tissue reactions and not quantitative assessments of systemic inflammatory cytokines after vaccination. We next tested whether each vaccine could induce *in vivo* Ab responses against influenza in mice. Indeed, the vaccinated mice produced IgG Abs against the HA subtype encoded by the vaccine plasmid, albeit with substantial variation in IgG titers (Figure S2A). The Abs were specific for the vaccine-encoded HA, and there was little cross-reactivity to other HA subtypes (Figure 1D). The minor cross-reactivity observed was predominantly directed against phylogenetically related HA subtypes (Figures 1A and 1D). Due to the observed differences in Abs elicited by DNA immunization, we next examined any correlation with protein expression levels. Indeed, HA-specific IgG titers correlated significantly with *in vitro* expression of the vaccine constructs (Figure S2B). That said, there were also substantial differences in IgG responses elicited by vaccines that were expressed at lower levels *in vitro.* For example, while both H2 and H15 were poorly expressed in supernatants, H15 induced high Ab titers upon immunization, while H2 did not (Figure S2B). In summary, it is likely that both the level of vaccine protein expression and the inherent immunogenicity of each HA subtype influenced vaccine-induced Ab levels.

Mice vaccinated with MIP1α-C_H_3-H1^PR8^ developed high levels of Abs against H1^PR8^ (Figure 1E) and were protected against a challenge with the PR8 virus [A/Puerto Rico/8/1934 (H1N1)] (Figure 1F). By contrast, none of the other 17 HA subtype vaccines induced H1^PR8^-specific Abs or protection, (Figures 1E and 1F). In summary, vaccination with MIP1α-targeted vaccines that bivalently expressed a single HA subtype elicited subtype-specific Ab responses and protection.

### Plasmid mixtures expressing either 17 or 18 HA subtypes induced cross-reactive Abs and protection against a non-included H1 virus

We next mixed the MIP1α-C_H_3 plasmids encoding each of the 18 HA subtypes into one vaccine bolus (MIP1α-C_H_3-MIX^18^). The 18 HA mixture contained H1 from A/California/07/2009 (H1N1) (H1^Cal07^) to enable a subsequent heterologous challenge with the PR8 influenza virus (H1^PR8^ and H1^Cal07^ share 82% sequence identity; Table S2A). We also prepared a vaccine mixture of 17 HA subtypes, in which H1 had been excluded completely (MIP1α-C_H_3-MIX^18ΔH1^), to assess broader heterosubtypic cross-protection. The mixtures contained equimolar concentrations of 1 μg of each vaccine plasmid.

Mice were vaccinated with the different plasmid mixtures, injected as a single bolus. Control mice received a non-targeted control plasmid mixture, in which the MIP1α targeting unit had been exchanged with an scFv specific for the hapten 4-hydroxy-3-iodo-5-nitrophenylacetic acid (NIP) (αNIP-C_H_3-MIX^18^)^19^ or mock vaccinations with saline. All the vaccine mixtures, including the αNIP-C_H_3-MIX^18^, induced significant levels of IgG specific for H1^Cal07^ after two vaccinations (Figure 1G). Inclusion of H1^Cal07^ in the mixture resulted in higher Ab levels, but not significantly higher than those induced by a mix vaccine in which H1^Cal07^ had been excluded (MIP1α-C_H_3-MIX^18ΔH1^) (*p* = 0.292). When tested against the heterologous H1^PR8^, all vaccine mixtures induced similar titers of cross-reactive Abs (Figure 1H). Abs against H1^PR8^ were skewed toward the IgG2a subclass (Figure 1I), consistent with previous observations of an IgG2a/Th1-skewing effect of the MIP1α targeting unit.^10^ Finally, regardless of whether the plasmid mixtures contained H1^Cal07^ or not, the mice that received an MIP1α-targeted mixture were significantly protected (80%–100% survival) against a lethal, heterologous challenge with the PR8 virus (Figure 1J). Importantly, targeting the vaccine to CCR1/3/5 with MIP1α significantly improved survival (Figure 1J) and weight loss (Figure S2C) compared with mock vaccination, whereas the non-targeted control mix vaccine did not. The mice suffered symptoms and weight loss prior to recovery, indicating a non-sterilizing mechanism of protection (Figure S2C).

Taken together, plasmid mixtures encoding either 17 or 18 MIP1α-targeted HA subtypes induced protective immune responses against heterologous PR8 virus, even though H1^PR8^ had not been included in the vaccine mixture. MIP1α targeting also enhanced protection against the PR8 virus when compared with the non-targeted control vaccine mixture.

### Plasmid mixtures prepared with either a homodimerization (C_H_3) or a heterodimerization (A/B) motif result in different vaccine proteins

**Figure 2:**
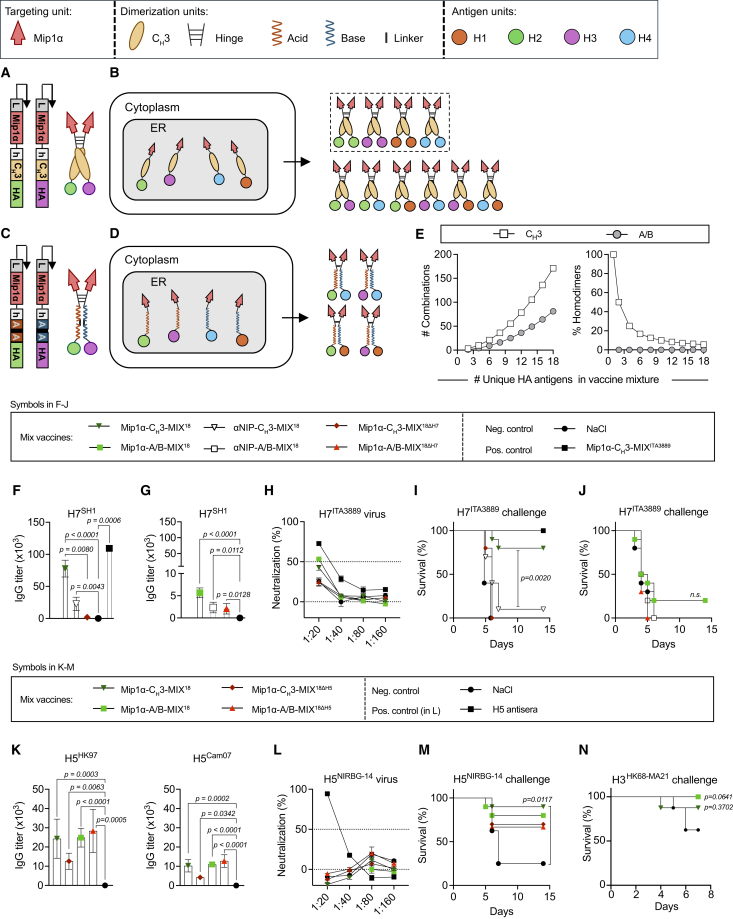
MIP1α-targeted plasmid mixtures induce cross-protection against heterologous H7N1 and H5N1 influenza viruses, as well as homologous H3N2 (A) Plasmid cassette (left) and schematic representation (right) of a vaccine protein in which two plasmid-encoded polypeptides associate via a C_H_3 homodimerization motif. (B) C_H_3-based homodimerization presumably results in dimers that stochastically combine plasmid-encoded polypeptides in the ER of a transfected cell, thereby generating dimers containing either two identical (boxed) or two different HA molecules. For simplicity, only four of the 18 different HA subtype polypeptides are shown. (C) Two plasmid cassettes (left) encoding A and B polypeptide chains, respectively, and a schematic representation (right) of a vaccine protein in which an A and a B chain dimerize via an A/B heterodimerization unit. (D) The A/B heterodimerization motif restricts heterodimer formation to pairing of polypeptides that express the A and B motifs, respectively. Therefore, only dimers that are monovalent for a given HA subtype are formed. For simplicity, only four of the 18 different HA subtype polypeptides are shown. HA subtypes were allocated to A and B chains to maximize the difference between any two HA subtypes associated with a single vaccine protein. (E) The number of different HA subtype combinations and HA subtype-bivalent molecules as a function of the number of HA subtypes included in C_H_3 (adapted from ref.^13^) and A/B plasmid vaccines. (F–J) In two separate experiments, BALB/c mice (*n* = 10/group, *n* = 4 for the Mip1α-C_H_3-H7^ITA99^ positive control) were vaccinated twice at weeks 0 and 5 with the indicated vaccines. (F and G) Five weeks after the boost, IgG responses were measured in individual mice against heterologous H7^SH1^ (mean ± SEM). (H) Pooled sera from groups of mice in both experiments in (F) and (G) were analyzed in a microneutralization assay against heterologous A/turkey/Italy/3889/1999 (H7N1) (H7^ITA3889^) influenza virus (the dashed line marks the 50% limit for positive neutralization, mean of technical triplicates ± SEM). (I and J) In both experiments, 5 weeks after the boost, mice were challenged with the heterologous H7^ITA3889^ virus (5×LD_50_) and monitored for survival. (K–M) BALB/c mice (*n* = 10/group) were vaccinated twice at weeks 0 and 4 with the indicated vaccines. (K) Five weeks after the boost, H5-specific serum IgG responses against homologous H5^HK97^ and heterologous H5^Cam07^ were measured in individual mice (mean ± SEM). (L) Sera pooled per group were analyzed in a microneutralization assay against the heterologous NIBRG-14 (H5N1) influenza virus. An antiserum against the H5N1 virus was included as a positive control (filled square). (The dashed line marks the 50% limit for positive neutralization. mean of technical triplicates ± SEM). (M) Five weeks after the boost, mice were challenged with the heterologous NIBRG-14 virus (5×LD_50_) and monitored for survival. (N) BALB/c mice (*n* = 8/group) were vaccinated twice at weeks 0 and 4 with the indicated vaccines. Five weeks after the boost, mice were challenged with homologous H3N2 influenza virus (1×LD_50_) and monitored for weight loss. Statistical significance was calculated using the Kruskal-Wallis with Dunn’s multiple comparisons test in (F), (G), and (K), or the Gehan-Breslow-Wilcoxon test in (I), (J), (M), and (N). Survival curves represent mice being euthanized at 20% weight loss as the humane endpoint. Only significant values are shown.

In the experiments to follow, we compared vaccine mixtures formulated with either the C_H_3 homodimerization unit^19^ or the A/B heterodimerization unit,^20^ the latter being based on a modified Fos-Jun zipper.^21^ These two formats differ with respect to how the X-shaped protein display HA subtypes. The C_H_3-based vaccine proteins randomly present two HA subtypes that might be either identical or different (Figures 2A and 2B). In contrast, the A/B-based vaccine proteins always displays two different HA subtypes on each arm of the vaccine proteins (Figures 2C and 2D).

In more detail, upon i.m. plasmid vaccination, vaccine-encoded polypeptides should be transported through the endoplasmic reticulum (ER) of transfected myocytes^22^ (Figures 2B and 2D). When using HA mix vaccines, a single myocyte is likely to be transfected simultaneously with several different plasmids that encode polypeptides carrying different HA subtypes.^13^^,^^20^ When using the C_H_3 homodimerization unit, any vaccine polypeptide containing a C_H_3 motif can presumably dimerize with any other C_H_3-containing polypeptide present^13^ (Figure 2B). Assuming stochastic and equivalent dimerization, a mixture of 18 different HA subtype-encoding polypeptides should yield 171 ([n(n+1)]/2) different dimeric vaccine proteins, of which 5.6% (1/n) would be homodimers displaying two identical HAs (Figure 2E). By contrast, with the A/B heterodimerization format, we allocated each HA subtype to either the A or B motif. The allocation was done in such a way that phylogenetically distant HA subtypes would be expressed in the same vaccine proteins (allocation in Table S1). This strategy was chosen to enhance the cross-coupling of BCRs specific for epitopes conserved between the HAs.^13^^,^^14^ The 18 HA subtypes, divided equally between the A or B motifs, should yield 81 (n_A_ × n_B_) different vaccine proteins that always display two different HA subtypes (Figure 2E). Thus, by comparing Ab responses elicited by the two plasmid mixtures encoding the two different formats, we might detect differences in cross-reactivity and protection resulting from differences in which HA subtypes are expressed within single dimeric vaccine proteins. Moreover, we should be able to detect any contribution from the presence of some X-shaped vaccine proteins displaying two identical HA subtypes as homodimers with the C_H_3 dimerization.

### Plasmid mixtures expressing 18 HA subtypes protected against a vaccine-mismatched H7 virus

To examine the potential breadth of cross-protection conferred by the vaccine mixtures, we next assessed whether the protection extended to a group 2 influenza virus of the H7 subtype (Figure 1A). We prepared plasmid mixtures for two separate experiments using either the C_H_3 or A/B motif. For each experiment, two different vaccine mixtures were compared: one in which all 18 HA subtypes were included (MIX^18^), and one in which the H7 derived from A/chicken/Italy/13474/1999 (H7N1) (H7^ITA99^) was excluded (MIX^18ΔH7^). MIP1α was used as the APC-targeting unit, while the αNIP scFv served as a non-targeted control. After two vaccinations, the groups of mice that had received a vaccine mixture including the H7^ITA99^ subtype mounted IgG responses against a heterologous H7 derived from A/Shanghai/1/2013 (H7N9) (H7^SH1^) (sequence identity of 96% for H7^ITA99^ vs. H7^SH1^; alignment in Table S2B) (Figures 2F and 2G). The mice that received the Mip1α-C_H_3-MIX^18^ developed particularly high levels of H7^SH1^-specific IgG, with a significant boost over the Mip1α-C_H_3-MIX^18ΔH7^ group (Figure 2F). A possible explanation is that the fraction of vaccine proteins with bivalent H7^ITA99^ display could suffice for induction of potent anti-H7^SH1^ Abs, due to cross-reactivity between the two subtypes (Figure 2F).^8^ This would also explain the improved anti-H7^SH1^ Ab levels after vaccination with Mip1α-C_H_3-MIX^18^ (Figure 2F), as compared with the lower titers observed with Mip1α-A/B-MIX^18^ (Figure 2G).

Despite high titers following vaccination with Mip1α-A/B-MIX^18^, the anti-H7 Abs could not neutralize heterologous A/turkey/Italy/3889/1999 (H7N1) (H7^ITA3889^) virus *in vitro* (identity of 98% for H7^ITA99^ vs. H7^ITA3889^; alignment in Table S2B) (Figure 2H). Still, the MIP1α-C_H_3-MIX^18^ vaccine conferred significant protection against a lethal challenge with the H7^ITA3889^ virus, while the other vaccine candidates did not (Figures 2I, 2J, S3A, and S3B). The results demonstrate that a plasmid mixture of 18 HAs can elicit cross-protective immune responses against a heterologous H7 virus, with a clear benefit for vaccine mixtures with C_H_3 homodimerization.

### Plasmid mixtures expressing 17 or 18 HA subtypes with MIP1α protected against H5 and H3 influenza viruses

To assess protection against an H5 virus, groups of mice were vaccinated with MIP1α-targeted HA vaccine mixtures that either included or excluded the H5 antigen from A/Hong Kong/483/97 (H5N1) (H5^HK97^). All vaccines induced Abs against homologous H5^HK97^ and heterologous H5^CAM07^ (from A/Cambodia/R0405050/2007) (Figure 2K) (sequence identity of 95% for H5^HK97^ vs. H5^CAM07^; alignment in Table S2C). Inclusion or exclusion of H5^HK97^ did not significantly impact the IgG titers against H5^HK97^ nor H5^CAM07^ (Figure 2K). A possible explanation is that, since H5 belongs to a cluster of phylogenetically related HAs that includes H1, H2, and H6 (Figure 1A), these related HA antigens included in the mixed vaccines could contribute to the induction of cross-reactive Abs against H5. Still, the Abs failed to neutralize the heterologous reassorted A/Vietnam/1194/2004 × Puerto Rico/8/1934 (H5N1) (NIBRG-14) virus *in vitro* (Figure 2L) (sequence identity of 96% for H5^HK97^ vs. H5^NIBGR−14^; alignments in Table S2C).

In a lethal challenge with the NIBRG-14 virus, the vaccine mixtures containing the heterologous H5^HK97^ antigen significantly reduced weight loss compared with mock-vaccinated controls (Figure S3C). For overall survival, only the mice that received the Mip1α-C_H_3-MIX^18^ were protected to significant levels over mock controls (Figure 2M). In conclusion, a plasmid mixture of 18 HA could confer cross-protection against a heterologous H5 virus. The C_H_3 and A/B dimerization motifs had roughly equal efficiency, with a small benefit from the C_H_3 dimerization unit when all 18 HA were included (Figures 2M and S3C). Similarly, vaccine mixtures with all 18 HA present and either the C_H_3 or the A/B dimerization motifs could offer equal protection against a homologous viral challenge with influenza H3N2 (Figures 2N and S3D).

### MIP1α targeting enhanced vaccine-induced cross-protection as compared with MHCII-targeting

**Figure 3:**
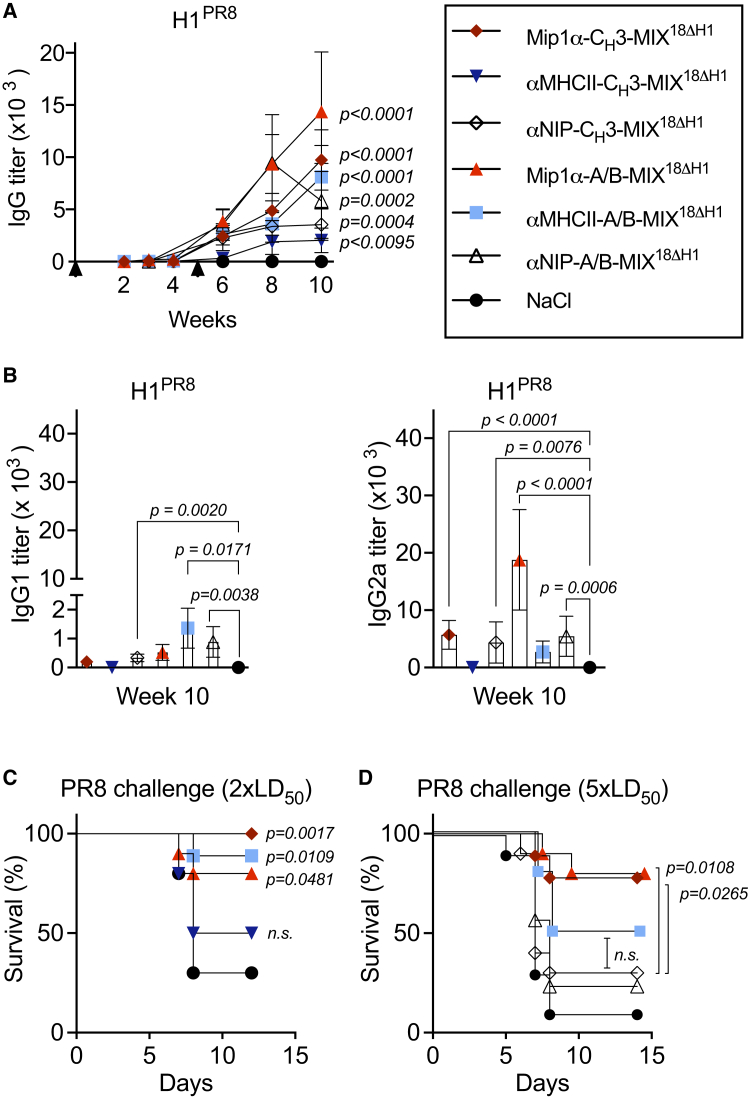
Mip1α targeting improves heterosubtypic cross-protection against the H1 virus BALB/c mice were vaccinated twice at weeks 0 and 5 (arrows) with the indicated plasmid mixtures. (A and B) Sera from individual mice (*n* = 9–20 mice/group) were analyzed by ELISA for H1^PR8^-specific antibodies. Shown are combined results from two identical experiments. (A) Total IgG was measured at weeks 2–10 (mean ± SEM). (B) IgG1 and IgG2a were measured at week 10 (mean ± SEM). (C and D) Five weeks after the boost, mice were challenged with PR8 influenza virus at either 2×LD_50_ (*n* = 9–10/group) (C) or 5×LD_50_ (*n* = 9–10/group) (D) and monitored for survival. Survival curves represent mice being euthanized at 20% weight loss as the humane endpoint. Weight loss data are shown in Figure S4.

In previous experiments with plasmid-encoded mixtures of 16 or 18 different HA subtypes, targeting APCs by an scFv specific for MHCII molecules showed low but significant cross-protection against viral challenge with PR8.^14^ We thus wanted to compare the relative efficacy of MIP1α and MHCII targeting of mixed vaccines. In addition, a comparison between the C_H_3 and A/B dimerization motifs was included. Mixtures of 17 HA subtypes in which the H1 subtype had been excluded (MIX^18ΔH1^) were used to maintain a challenge model for heterosubtypic protection against the H1 virus PR8. To measure the effect of APC targeting, we included non-targeted (αNIP) control mixtures for each dimerization motif. After two vaccinations, all vaccine groups had mounted cross-reactive IgG responses against the H1^PR8^ subtype not included in the vaccine mixtures (Figure 3A). IgG1 responses against H1^PR8^ were low, and only the αMHCII-A/B-MIX^18^^Δ^^H1^ and αNIP-targeted groups showed significant increases over NaCl-vaccinated mice (Figure 3B). Both MIP1α-targeted vaccine mixtures significantly increased anti-PR8 IgG2a over mock-vaccinated controls (Figure 3B), in line with previous observations of Th1 skewing with Mip1α.^10^^,^^15^

Upon a viral challenge with a lethal dose (LD) of the heterosubtypic PR8 virus (2×LD_50_), the MIP1α-targeted vaccine mixtures with either A/B or C_H_3-based dimerization motifs significantly protected mice. For the MHCII-targeted vaccines, the mixture using A/B heterodimerization protected mice, whereas C_H_3 homodimerization did not (Figure 3C). To further gauge vaccine-induced protection, mice were, in a second experiment, challenged with a higher dose of influenza PR8 virus (5×LD_50_). Against this higher dose, only the MIP1α-targeted HA mixtures conferred significant protection, while the MHCII-targeted and non-targeted vaccines did not protect (Figure 3D). The mice suffered weight loss prior to recovery, consistent with non-sterilizing immunity (Figure S4).

In sum, MIP1α-targeted plasmid mixtures expressing 17 HA antigens induced cross-reactive, IgG2a-skewed Ab responses and heterosubtypic cross-protection against the H1 virus PR8. Moreover, MIP1α was superior to αMHCII as a targeting unit, while A/B and C_H_3 dimerization motifs were of roughly equal efficacy.

### Heterodimerization affected strain-specific but not cross-reactive responses

**Figure 4:**
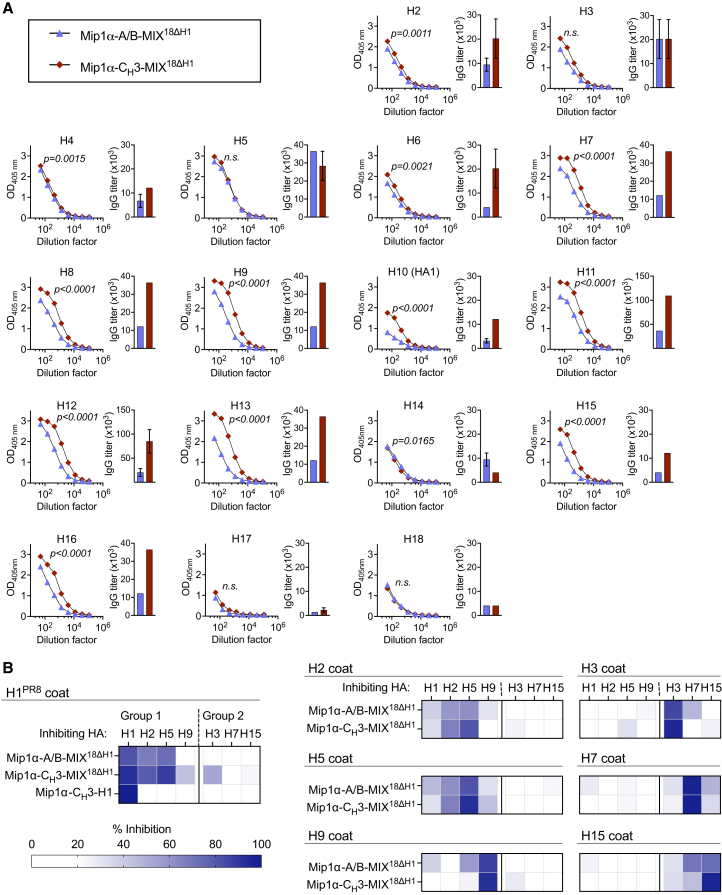
Mip1a-targeted vaccine mixtures induce antibodies that bind all HA subtypes and cross-reactive antibody responses that bind phylogenetically related HA subtypes BALB/c mice (*n* = 10/group) were vaccinated twice at weeks 0 and 5 with the indicated vaccines. (A) Five weeks after the boost, pooled sera were analyzed for IgG specific for H2–H18 by ELISA (mean ± SEM of technical triplicates in bars). (B) Pooled sera from mix-vaccinated mice were analyzed for antibody cross-reactivity between different HA subtypes. Sera were incubated with soluble H1, H2, H5, or H9 (group 1 HA subtypes) or H3, H7, or H15 (group 2 HA subtypes) prior to analysis for binding to each of the same HA immobilized on the solid phase as a coat in ELISA. Pooled sera from mice immunized with Mip1a-C_H_3-H1^PR8^ were included in the experiment with the H1^PR8^ coat. Cross-reactivity was calculated as percent inhibition, measured as the reduction in signal compared to non-inhibited control samples. Statistical significance in (A) was calculated by an unpaired *t* test comparing the AUC of the reported curves.

The experiments outlined above have repeatedly demonstrated cross-reactivity to non-included HA subtypes when vaccinated with HA-encoding plasmid mixtures. However, they also indicate differences in Ab generation and vaccine-induced protection between vaccines equipped with either the homodimeric C_H_3 or the heterodimeric A/B motifs. We therefore wanted to compare strain-specific and cross-reactive Ab responses in groups of mice vaccinated with either MIP1α-A/B-MIX^18ΔH1^ or MIP1α-C_H_3-MIX^18ΔH1^. First, both the A/B- and C_H_3-containing plasmid mixtures induced HA-specific responses against all HA subtypes included in the vaccine mixtures (Figure 4A). Second, the mixture containing the C_H_3-based homodimerization domain yielded significantly higher Ab responses against 13 of the 17 included HA subtypes compared with the equivalent mixture containing the A/B-based heterodimerization domain (Figure 4A). These results indicate that even a small fraction of homodimers in C_H_3-based mixtures can increase HA subtype-specific responses.

To assess cross-reactivity induced by the two types of HA subtype mixtures, pooled sera from mice vaccinated with either MIP1α-C_H_3-MIX^18ΔH1^ or MIP1α-A/B-MIX^18ΔH1^ were analyzed using a competitive ELISA. Specifically, diluted sera from vaccinated mice were pre-absorbed with soluble recombinant H1^PR8^, H2, H5^HK97^, H9, H3, H7^SH1^, or H15 prior to assessment of Ab binding to the same recombinant HA subtypes immobilized on microtiter plates. Both vaccines induced cross-reactive Abs that bound phylogenetically related HA from group 1 (H1^PR8^, H2, H5^HK97^) and group 2 (H7^SH1^/H15) (Figure 4B). However, heterodimeric display of phylogenetically distant HA on individual vaccine protein dimers obtained with the MIP1α-A/B-MIX^18ΔH1^ vaccine did not substantially broaden cross-reactivity against the tested HA subtypes compared with the random dimerization obtained with the MIP1α-C_H_3-MIX^18ΔH1^ vaccine. As a control, serum elicited by bivalent MIP1α-C_H_3-H1^PR8^ induced anti-H1^PR8^ Abs that did not cross-react with other HA subtypes in this assay (Figure 4B). In summary, vaccination with the HA subtype mixtures induced HA cross-reactive Abs, but the breadth of cross-reactivity was limited to phylogenetically related HA subtypes. These results were similar regardless of the dimerization motif (A/B or C_H_3) used.

### MIP1α-targeted plasmid mixtures induced cross-reactive B and T cell responses to heterologous HA

**Figure 5:**
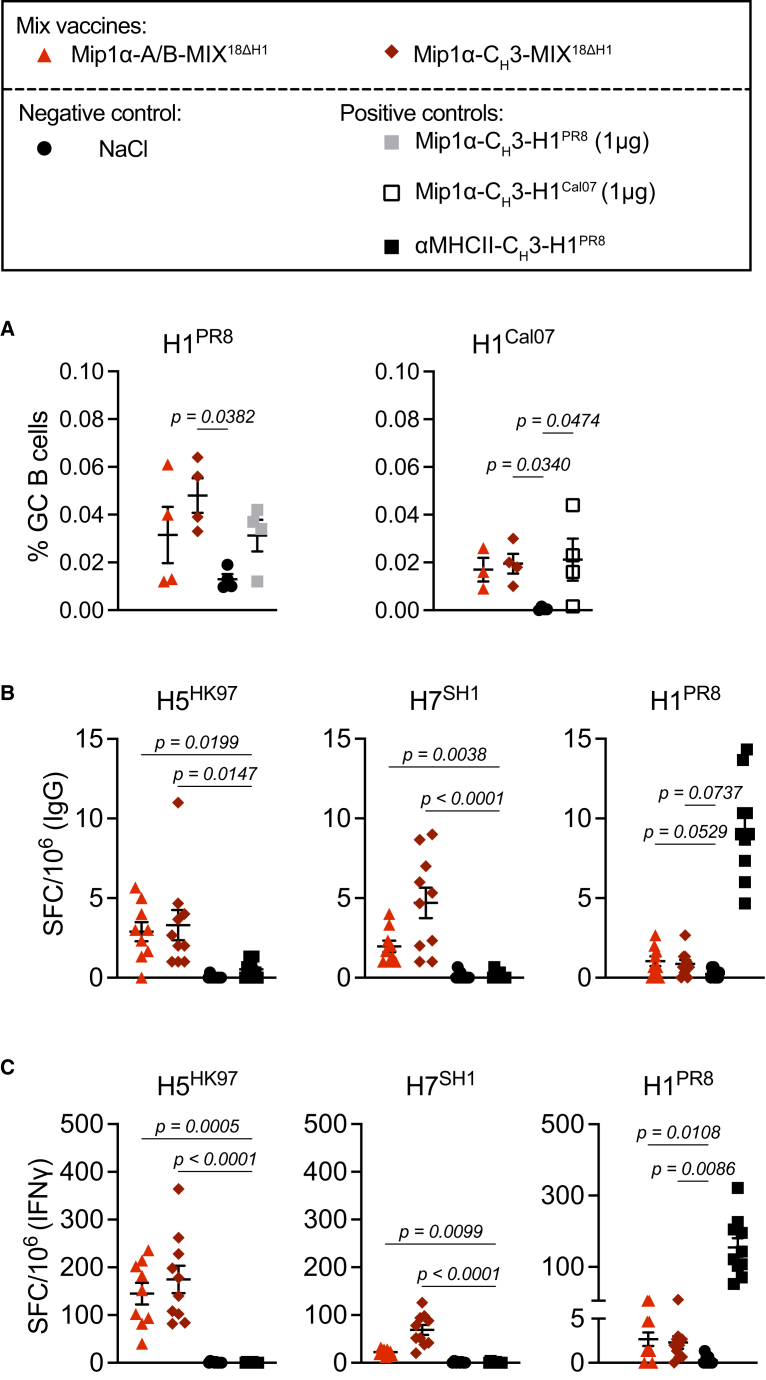
Mip1α-targeted vaccine mixtures induce cross-reactive and HA-specific B and T cell responses (A) BALB/c mice were vaccinated twice at weeks 0 and 5 with the indicated vaccines (*n* = 3–4/group). Eight days after the boost, single-cell suspensions of draining lumbar and para-aortic LNs were analyzed by flow cytometry for GC B cells (CD38^−^GL7^+^) reactive to 5×HIS-tagged H1^PR8^ and H1^Cal07^ (gating strategy shown in Figure S5). (B and C) Mice (*n* = 9–10/group) were vaccinated at weeks 0 and 5 with the indicated vaccines. BM and spleens were harvested five weeks after the boost. The number of spot-forming cells (SFC) per 1 million added cells is depicted. (B) BM single-cell suspensions were analyzed for IgG production by ELISpot against immobilized full-length HA from homologous H5^HK97^, heterologous H7^SH1^, and non-included H1^PR8^. (C) Splenic single-cell suspensions were analyzed for IFNγ production by ELISpot after stimulation with the same HA proteins as in B. Statistical significance was calculated using the Kruskal-Wallis test with Dunn’s multiple comparison.

Since we observed that MIP1α-targeted HA mixtures could induce cross-reactive Abs against the non-included H1 subtype (Figures 1H and 4B), we evaluated H1-reactive germinal center (GC) B cells in mice vaccinated with either MIP1α-A/B-MIX^18ΔH1^ or MIP1α-C_H_3-MIX^18ΔH1^. Vaccination with 1 μg of homodimeric MIP1α-C_H_3-H1 (either H1^PR8^ or H1^Cal07^), corresponding to the amount of one HA subtype plasmid in the vaccine mixtures, served as a positive control. The results showed that the draining lymph nodes (dLNs) of mice vaccinated with MIP1α-targeted vaccine mixtures had detectable H1-reactive GC B cell responses by flow cytometry, but only the MIP1α-C_H_3-MIX^18ΔH1^ group reached statistical significance compared with mock-vaccinated control mice (Figure 5A; gating strategy and populations are shown in Figure S5). In a second experiment, the two vaccine mixtures induced bone marrow (BM) plasma cells (PCs) that secreted IgG against the vaccine-homologous H5^HK97^ and heterologous H7^SH1^ antigens (Figure 5B). We also detected PCs that secreted cross-reactive IgG binding the non-included H1^PR8^ subtype, although these levels did not reach statistical significance (*p* = 0.0737 for MIP1α-C_H_3-MIX^18ΔH1^ and *p* = 0.0529 for MIP1α-A/B-MIX^18ΔH1^).

As for T cell responses, both MIP1α-targeted vaccine mixtures induced IFNγ-producing T cells against homologous H5^HK97^, heterologous H7^SH1^, and the non-included H1^PR8^ subtype (Figure 5C). In summary, both A/B- and C_H_3-based MIP1α-targeted vaccine mixtures induced GC B cells, PCs, and T cell responses.

### Abs mediate cross-protection against an H1 virus not included in the vaccine mixtures

**Figure 6:**
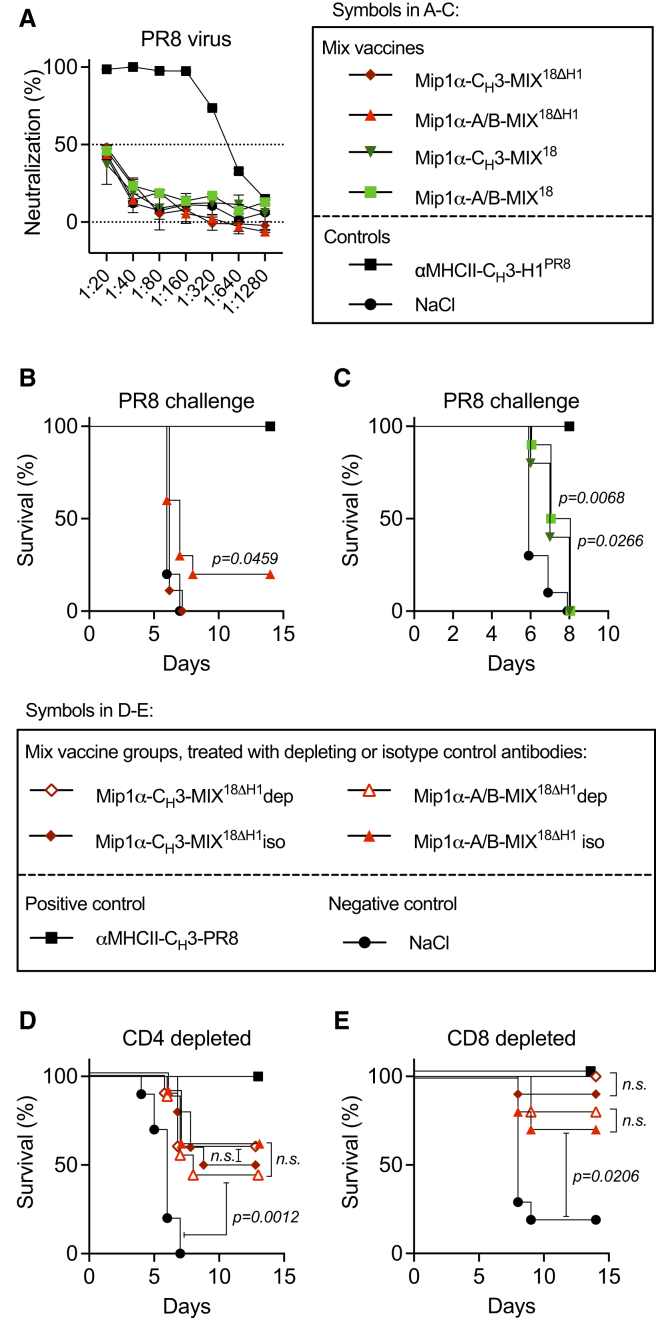
Cross-reactive antibodies contribute to protection against the non-included H1 PR8 virus (A–C) BALB/c mice (*n* = 10/group) were vaccinated twice at weeks 0 and 5 with the indicated vaccines. Five weeks after the second vaccination, mice were exsanguinated, and equal amounts of serum were pooled per group. (A) Microneutralization assay against PR8 virus. The dashed line marks the 50% threshold for positive neutralization. Mean of technical triplicates ± SEM. (B and C) In two separate experiments, 200 μL of pooled serum was transferred i.v. to naive BALB/c mice (*n* = 10/group). The next day, mice were challenged with PR8 influenza (5×LD_50_) and monitored for survival. (D and E) In two separate experiments, BALB/c mice (*n* = 10/group) were vaccinated twice at weeks 0 and 5 with the indicated vaccines. Five weeks after the second vaccination, mice were challenged with a 5×LD_50_ dose of PR8 influenza virus and monitored for weight loss (mean ± SEM), with a humane endpoint of 20% weight loss. On days −2, 0, and +2 relative to infection, mice were injected with either anti-CD4 (D) or anti-CD8 (E) depleting mAb, or an isotope-matched control, as indicated. Statistical significance was calculated using the Gehan-Breslow-Wilcoxon test. All survival curves represent mice being euthanized at 20% weight loss (humane endpoint).

Since the MIP1α-targeted vaccine mixtures induced both cross-reactive Abs and T cells, we next assessed the independent contribution of Abs to protection against influenza. We first evaluated pooled sera from mice vaccinated twice with MIP1α-C_H_3-MIX^18ΔH1^, MIP1α-A/B-MIX^18ΔH1^, MIP1α-C_H_3-MIX^18^, or MIP1α-A/B-MIX^18^ (H1 from Cal07 was included in the latter two) in a microneutralization assay against the PR8 virus. None of the vaccine mixtures included Abs capable of neutralizing PR8, whereas the positive control vaccine αMHCII-C_H_3-H1^PR8^ did (Figure 6A). Despite this, passive transfer of 200 mL of sera from vaccinated mice to naive mice prior to a lethal PR8 challenge demonstrated a small but significant delay in mortality following transfer of sera from mice vaccinated with MIP1α-A/B-MIX^18ΔH1^ (Figure 6B), MIP1α-C_H_3-MIX^18^, and MIP1α-A/B-MIX^18^ (Figure 6C), but not MIP1α-C_H_3-MIX^18ΔH1^.

The results indicated protection mediated by non-neutralizing Ab effector functions, which include FcγR-mediated natural killer (NK) cell activation and lysis of infected cells.^23^^,^^24^ Indeed, both αMHCII-targeted and MIP1α-targeted vaccine mixtures lacking PR8 induced PR8-reactive Abs capable of activating NK cells (Figure S6). However, NK cell activity alone could not explain the improved protection conferred by MIP1α-targeting (Figure 3D), as targeting toward MHCII was most effective in inducing Ab-mediated NK cell activation.

Serum transfer represents a large dilution (approximately 1:10) of Abs in recipient mice, which may explain the weak effect compared to that observed in vaccinated mice. Therefore, as an alternate strategy, we depleted vaccinated mice of T cells prior to the influenza PR8 virus challenge. This procedure should remove T cell-mediated protection while leaving vaccine-induced Abs intact. Mice were vaccinated twice with MIP1α-C_H_3-MIX^18ΔH1^, MIP1α-A/B-MIX^18ΔH1^, or the αMHCII-C_H_3-H1^PR8^ positive control vaccine. In two separate experiments, vaccinated mice were depleted of either CD4^+^ or CD8^+^ T cells by monoclonal Ab (mAb) injections prior to and during a lethal challenge with PR8 influenza virus. Depletion efficacy was confirmed by flow cytometry (Figures S7A and S7B). Compared with vaccinated control groups treated with an isotype-matched mAb, neither depletion of CD4^+^ T cells (Figures 6D, S7A, and S7C) nor depletion of CD8^+^ T cells (Figures 6E, S7B, and S7D) diminished mixture vaccine-induced protection against PR8 virus. Collectively, these results suggest that the cross-reactive Abs induced by MIP1α-targeted vaccination with HA mixtures contribute to heterosubtypic protection against the non-included PR8 virus. However, these experiments do not rule out an additive protective role of T cells in cross-protection.

## Discussion

DNA vaccines encoding mixtures of 17 or 18 different HA subtypes targeted to APC induced broadly cross-reactive Abs, T cells, and heterologous protection against influenza viruses, including protection against virus subtypes or strains not included in the mixtures. These results strengthen our hypothesis that the display of different HA subtypes on each arm of dimeric vaccine proteins can skew Ab responses toward conserved epitopes shared between different HAs, a phenomenon we have named valency-based immunoselection.^13^^,^^14^ In line with our findings, others have shown that nanoparticle vaccines displaying different HA antigens from H1^25^ or HA from seasonal H1, H3, and influenza B viruses,^26^ induced broad B cell responses and protection. An important observation from these latter studies was that broad reactivity was enhanced only when the different antigens were combined on a single nanoparticle, as compared to “cocktail” delivery of several particles, supporting our hypothesis regarding the importance of BCR cross-linking. More recently, another study demonstrated that immunization with a pool of 20 mRNA lipo-nanoparticles, each encoding one of the 20 known HAs from IAV and influenza B, induced cross-protective Abs.^27^ Similar to our past^13^^,^^14^ and current experiments, protection against vaccine-mismatched viruses was mediated by non-neutralizing Abs, but the underlying mechanism for induction of such Abs was not demonstrated.^27^ Taken together, DNA and mRNA emerge as useful formats for simultaneous, valency-based delivery of many HA subtypes.

Previously, we have demonstrated that plasmid mixtures containing an MHCII-specific targeting unit induced significant protection against H1 viruses that were not included in the vaccine mixture after three vaccinations.^13^^,^^14^ Here, we show in a head-to-head comparison that targeting the vaccine mixture to CCR1/3/5 by use of the chemokine MIP1α (CCL3) substantially improved cross-protection compared with targeting to MHCII. Moreover, APC-targeting by MIP1α induced cross-protection against non-included H1 viruses after just two vaccine doses.^13^^,^^14^ Importantly, the prospect of translating MIP1α HA mix vaccines to humans is promising, as human MIP1α (LD78b) has been shown to enhance immune responses in mice,^16^ and since LD78b was used in promising phase 1/2a studies in women with cervical intraepithelial neoplasia^17^ and advanced cervical cancer.^18^ That said, for progression to larger animals and humans, the strategy used here of needle injection followed by electroporation (EP) is likely not feasible for prophylactic mass vaccination. A gentler strategy would be to use jet delivery, and we are presently using this for an ongoing clinical Phase I trial to evaluate safety and immunogenicity following HLAII-targeted vaccination against influenza H7N9 (NCT06046092).

MIP1α is specific for CCR1/3/5,^28^ which are expressed on both cDC1 and cDC2, as well as on macrophages and monocytes. APC-targeting by MIP1α has previously been shown to skew immune responses toward Th1/IgG2a,^10^^,^^15^ resulting in the induction of strong CD8^+^ T cell responses.^10^^,^^29^ In mice, the IgG2a subclass is associated with non-neutralizing effector mechanisms against influenza, including FcγR-mediated mechanisms such as ADCC,^23^ complement-dependent lysis,^24^ and Ab-dependent phagocytosis.^30^ Others have identified ADCC as a significant mechanism for viral clearance by Abs binding conserved epitopes on the HA stem^31^^,^^32^ and head.^33^^,^^34^ In humans, broadly reactive ADCC Abs were implicated in mitigating disease burden during the 2009 H1N1 “Swine Flu” pandemic.^35^ Our MIP1α-targeted HA mixtures induced IFNγ-producing T cells (Th1) and skewed anti-HA Abs toward IgG2a. Indeed, the cross-reactive Abs in our experiments could not neutralize viruses, but they could induce FcγR-mediated effector functions, as measured by NK cell activation (Figure S6). Furthermore, serum transfer from vaccinated to naive mice conferred weak protection against a viral influenza challenge. Vaccine-induced protection also persisted after depletion of CD8^+^ or CD4^+^ T cells, supporting the importance of Abs. However, we could not explain the increased efficacy of Mip1α-targeting over targeting to MHCII by non-neutralizing Abs alone, as MHCII-targeting yielded the highest levels of NK cell activation (Figure S6). Furthermore, as the protection observed following serum transfer (Figures 6B and 6C) or T cell depletion (Figures 6D and 6E) was reduced compared to that observed in the presence of an intact immune system (Figures 2I, 2J, 2M, and 2N), it is likely that cross-reactive Abs and T cells acted in concert to provide the observed cross-protection.

We have here compared plasmid mixtures constructed with the C_H_3 homodimerization and the A/B heterodimerization motifs, respectively. The C_H_3-based domain results in a much larger repertoire of dimeric proteins expressing two different HAs, and in addition, a fraction of the dimeric proteins will express two identical HAs.^13^ The A/B-based motif exclusively results in heterodimeric proteins that always express two different HAs. By careful selection of which HA subtypes are expressed with either the A or B chains, homology between the two HAs expressed in single A/B vaccine proteins may be reduced, a strategy used previously^14^ and herein. Mixtures using the A/B motif result in a lower number of different vaccine proteins compared to use of the C_H_3 motif. Given these differences, it was surprising to observe that A/B- and C_H_3-based mixtures targeted by MIP1α resulted in similar patterns of Ab cross-reactivity. We did, however, observe a benefit of the C_H_3 dimerization motif for 18 HA mixtures in heterologous challenge, pointing to a potential benefit of dimerization of identical or phylogenetically clustered HAs.

The MIP1α-targeted vaccine mixtures that encoded all HA subtypes except H1 induced Abs that cross-reacted with H1. Most likely, similar but not identical HA subtypes could occasionally assemble into single dimeric vaccine proteins, and these could play a pivotal role in the induction of such cross-reactive Abs. The results suggest that vaccine proteins deliberately composed of similar HA subtypes may improve elicitation of broadly reactive Abs to conserved epitopes within that selected group of HAs. It is indeed possible to construct more homogeneous HA mixtures than those used herein, for example, mixtures of multiple strains from just a single HA subtype.

In conclusion, the MIP1α-targeted HA plasmid mixture vaccine results in the simultaneous display of many HA subtypes containing several shared conserved epitopes. Such vaccination elicits cross-reactive Ab responses against conserved epitopes and provides protection against influenza viruses not previously seen by the vaccinated mouse. The strategy could be translated into a broadly protective influenza A vaccine for humans. The platform can likely be adapted to other pathogens where antigen evolution and escape by immunoselection is issues, such as the spike protein of the severe acute respiratory syndrome coronavirus 2 (SARS-CoV-2) virus.

## Materials and methods

### Construction of APC-targeted DNA vaccines

The DNA vaccines were human cytomegalovirus (CMV) promotor-based pLNOH2 plasmid vectors^36^ encoding a polypeptide chain consisting of a targeting, a dimerization, and an antigen unit.^11^^,^^19^ The targeting unit consisted of either the MIP1α,^28^ a single-chain fragment variable (scFv) specific for the MHCII molecule I-E^d^ of the BALB/c H-2^d^ haplotype (αMHCII),^7^^,^^19^ or a scFv specific for the hapten NIP (αNIP, non-targeted control),^7^^,^^19^ as previously described. The dimerization units consisted of either the hinge exons 1 and 4 and the C_H_3 domain of human IgG3 (C_H_3),^19^ or the hinge exon 1 and leucine zipper alpha helixes enriched with either acidic (A) or basic (B) amino acids (aa).^20^ The HA subtypes used as antigenic units are specified in Table S1, and for each, the full ectodomain was inserted (aa 18–541 of group 1 constructs by PR8 numbering, except for H5, where aa 18–532 was used; for group 2 antigens, aa 19–536 was used by H7 numbering). The vaccine plasmid cassettes contain specific restriction sites flanking the targeting unit (*Bsm*I and *Bsi*WI) and the antigenic unit (*Sfi*I and *Sfi*I). The vaccine plasmids encoding the 18 HA subtypes linked to MIP1α, αMHCII, or αNIP by either a C_H_3 or A/B dimerization motif were thus constructed from existing αMHCII-A/B-HA and αMHCII-C_H_3-HA plasmids by exchanging either the targeting or antigenic units.^7^^,^^13^^,^^14^

### Multiple sequence alignment of HA proteins and generation of a phylogenetic tree

The phylogenetic tree of the HA proteins used in the vaccines was generated from aa sequences using Clustal Omega for multiple alignment and the neighbor-joining method without distance correction (URL: https://www.ebi.ac.uk/jdispatcher/phylogeny/simple_phylogeny)^37^ and visualized using the TreeDyn 198.3 online tool (URL: http://www.phylogeny.fr/).^38^ HA protein alignments were performed with the NCBI blastP suite’s multiple alignments tool (URL: https://blast.ncbi.nlm.nih.gov/Blast.cgi).^39^

### Mice, cell lines, recombinant proteins, and viruses

Six- to twelve-week-old BALB/c mice (Taconic, Ejby, Denmark, and Janvier, le Genest-Saint-Isle, France) were housed under minimal disease conditions at the Department of Comparative Medicine, Oslo University Hospital, Rikshospitalet. The Norwegian Food Authority approved all experiments. Human embryonic kidney cells (HEK293E) and Madin-Darby Canine Kidney (MDCK) cells were from the American Type Culture Collection (ATCC, Manassas, USA). Recombinant (rec.) HAs were ordered from Sino Biological (Table S1). Live virus A/Puerto Rico/8/1934 (Mt. Sinai sub-strain) (H1N1) (PR8) was a kind gift from Dr. Anna Germundsson at the National Veterinary Institute, Norway. The A/turkey/Italy/3889/1999 (H7N1) virus^8^ and the reassortant virus NIBRG-14 (Vietnam/1194/2004 x Puerto Rico/8/1934) (H5N1), expressing H5 from A/Vietnam/1194/2004 (H5N1)^40^ were kind gifts from Prof. Rebecca Cox at the University of Bergen, Norway. The A/turkey/Italy/3889/1999 (H7N1) virus was mouse-adapted by serial passage prior to use.^8^ The A/Hong Kong/1/1968(H3N2)-MA21 virus (NR-28634) was kindly provided by BEI Resources.

### *In vitro* expression and detection of vaccine proteins by ELISA

HEK293E cells were transfected at approximately 80% confluence in 24-well plates (Corning, NY, USA) and cultured overnight (ON) in complete medium at 37°C/5% CO_2_. On the day of transfection, the medium was replaced with FreeStyle 293 serum-free medium (Life Technologies). Cells were transfected with polyethylenimine (PEI) complexed with 1 μg of DNA plasmids for the C_H_3-dimerized vaccines. For A/B dimerized vaccines, cells were transfected with PEI complexed with 0.5 μg DNA plasmids of paired A and B plasmid counterparts (1 μg in total). After 3 days, supernatants were harvested and centrifuged at 3,000 rpm for 5 min before further analysis. Supernatants were added to 96-well plates (Costar 3590, Corning, NY, USA) pre-coated with a mAb specific for the C_H_3 domain of hγ3 chains (MCA878G, 1 μg/mL, AbD Serotec, Hercules, CA) or NIP-conjugated BSA (produced in-house). Secreted vaccine proteins were detected with a biotinylated mAb specific for C_H_3 of hγ3 chains (HP6017, B3773, 1:3000, Sigma-Aldrich), biotinylated anti-MIP1α (MAB450, 1 μg/mL, R&D systems), or a biotinylated anti-A/B mAb (2H11, 1 μg/mL, a kind gift from Dr. Ellis L. Reinherz), followed by a streptavidin-alkaline phosphatase conjugate (1:3000, Calbiochem). Plates were developed with phosphatase substrate (P4744-10G, 1 mg/mL, Sigma-Aldrich) and read at 405 nm after 1 h using a Tecan Sunrise plate reader (Tecan, Mannedorf, Switzerland) with Magellan v5.03 software.

### Vaccination and serum sampling

DNA plasmid vaccines were purified using the EndoFree plasmid Megaprep kit (12381, Qiagen, Hilden, Germany) and dissolved in sterile 0.9% sodium chloride (NaCl) (B.Braun, Melsungen, Germany). Mice were anesthetized with a cocktail of Zoletil Forte (250 mg/mL, Virbac France), Rompun (20 mg/mL, Bayer Animal Health GmbH), and fentanyl (50 μg/mL, Actavis Germany) (ZRF) by intraperitoneal injection at 0.1 mg/10 g body weight. The C_H_3-dimerized HA-mix vaccines contained 1 μg of each HA-encoding plasmid per mouse. The A/B dimerized HA-mix vaccines contained 1 μg of each HA-encoding plasmid coupled to either the A or B dimerization motif (Table S1), resulting in a total DNA dose corresponding to the C_H_3-dimerized mixtures, thus equalizing chemotactic effects of MIP1α-targeting. All DNA plasmids were diluted in 0.9% NaCl to a total injection volume of 100 μL per mouse. After shaving the vaccination area, vaccines were administered as intramuscular (*i.m.*) injections of 50 μL into each quadriceps muscle, immediately followed by needle EP with an AgilePulse (Elgen, Inovio Biomedical Co., Blue Bell, PA, USA). Mice were monitored for signs of adverse effects of sedation or vaccination for 24 h after the procedure. Blood samples (100 μL) were collected by saphenous venous puncture. Larger volumes were obtained by cardiac puncture under sedation prior to euthanasia. Serum was separated by centrifugation twice at 15,000 rpm for 10 min and stored at −20°C until analysis.

Muscle biopsies were collected 24 h post-vaccination from the injection site and fixed in formalin before embedding in paraffin. Slices were mounted on slides, deparaffinized, and stained with hematoxylin and eosin (H&E). Tissue processing was performed by the Section of Histology, Department of Pathology (Rikshospitalet, Oslo, Norway). Images were acquired on an Olympus VS200 slide scanner at 20× magnification.

### Serum Ab ELISA

ELISA 96-well plates (Costar 3590) were coated with HA protein (0.5 μg/mL, Sino Biological, or in-house) and incubated ON at 4°C. Coated plates were blocked prior to the addition of 3-fold serially diluted mouse sera, starting at a 1:50 dilution. Total IgG was detected with alkaline phosphatase (AP)-conjugated anti-mouse IgG (A1418, 1:5000, Sigma-Aldrich). Plates were developed as described above. IgG1 and IgG2a subclass Abs were detected with biotinylated anti-mouse IgG1 (553500, 1.0 μg/mL, BD Pharmingen) or biotinylated anti-mouse IgG2a (553502, 1.0 μg/mL, BD Pharmingen), followed by horseradish peroxidase (HRP)-conjugated streptavidin (1:5000, Southern Biotech) and TMB substrate (CL07-1000ML, Merck Millipore). Development was stopped after approximately 10 min with 100 μL of 1M hydrogen chloride (HCl). Optical density 450 (OD_450_) was measured as described above. Endpoint titers were determined using a cutoff value > [mean + (5 × SEM)] of samples from mice vaccinated with NaCl.

### HA inhibition assay

Mice were vaccinated twice (weeks 0 and 5). Five weeks after the boost, mice were anesthetized, and blood was harvested by cardiac puncture. As previously described,^14^ pooled sera were assayed for HA-specific IgG titers on ELISA plates coated with rec. HA from group 1 (H1[PR8], H2, H5[HK97], and H9) and group 2 (H3, H7[SH1], and H15) (0.5 mg/mL, Sino Biological). First, the serum dilution that yielded a signal of approximately OD_405nm_ 0.5 after 1 h was determined for each vaccine group on each HA coat. Next, serum samples were diluted accordingly and pre-incubated with one of the rec. HA (5 mg/mL) for 2 h at room temperature (RT) on a vertical shaker. Control samples were prepared without the rec. HA inhibitor. The samples were analyzed in technical triplicates on ELISA plates coated with each rec. HA. Detection of total IgG was performed as described above. The average background signal was determined from sera of mice vaccinated with saline and subtracted from sample wells. Inhibition was reported as the fraction of signal loss after pre-incubation with the inhibiting HA, compared to non-inhibited serum controls on each HA coat.

### Viral challenge

Anesthetized mice were infected by intranasal (*i.n.*) administration of live virus diluted in sterile PBS (10 μL per nostril). Viruses were diluted in PBS to either 1×LD_50_, 2×LD_50_, or 5L×D_50_ infectious doses. Mice were weighed daily and euthanized by cervical dislocation upon reaching 80% of pre-infection weight, or at the end of the experiment, as required by The Norwegian Food Authority.

### Detection of H1-reactive GC B cells

BALB/c mice were vaccinated twice (weeks 0 and 5). H1-reactive GC B cells were detected as previously described.^41^ Briefly, 1 week after the boost, para-aortic and lumbar dLNs were harvested, and single-cell suspensions were prepared using the GentleMACS dissociator (Miltenyi Biotech). Cells were blocked with 50% inactivated rat serum and stained with anti-CD3 (17A2, Tonbo Biosciences) (dump gate), anti-B220 (552771, BD Biosciences, Franklin Lakes, NJ, USA), anti-GL7 (144603, Tonbo Biosciences), anti-CD38 (102718, Tonbo Biosciences), and 6×His-tagged rec. H1 PR8 or Cal07 with a phenylalanine substitution for tyrosine at position 98 (Y98F) (purified in-house^41^ and detected with anti-6×His mAb [ab133714, Abcam, Cambridge, England]). Stained samples were fixed in 4% paraformaldehyde and run on an Attune NxT flow cytometer. Data were analyzed with FlowJo software (v7.6) (TreeStar, Ashland, OR).

### IFNγ and IgG enzyme-linked immunosorbent spot (ELISpot) assays

BALB/c mice were vaccinated twice (weeks 0 and 5) and euthanized 5 weeks after the boost. For the IFNγ ELISpot assay, spleens were harvested and prepared using the GentleMACS dissociator (Miltenyi Biotec), followed by incubation with tris-buffered ammonium chloride (ACT) erythrocyte lysis buffer for 5 min on ice. Single-cell suspensions (5.0 × 10^5^ cells/well) were seeded in technical triplicates for each mouse onto ELISpot plates pre-coated with anti-IFNγ (Mouse IFN-γ ELISpot PLUS [ALP] kit; Mabtech AB; Nacka Strand, Sweden). Cells were then stimulated with full-length rec. H1^PR8^, H5^HK97^, or H7^SH1^ (10 μg/mL, Sino Biological) for 18–20 h at 37°C in 5% CO_2_. Staining and development were performed according to the manufacturer’s protocol. For the IgG ELISpot assay, BM was harvested from the femur and tibia. Single BM cell suspensions of 5.0 × 10^5^ cells/well were seeded in technical triplicates for each mouse onto MultiScreen HTS filter plates (MSIPS4510; Millipore; Tullagreen, Ireland) pre-coated with recombinant H1^PR8^, H5^HK97^, or H7^SH1^ (10 μg/mL, Sino Biological) and incubated for 18–20 h at 37°C in 5% CO_2_. Spots were detected with AP-conjugated anti-mouse IgG (A1418, 1:5000, Sigma-Aldrich) and developed using phosphatase substrate (P4744-10G, 1 mg/mL, Sigma-Aldrich). All plates were analyzed with the CTL-ImmunoSpot analyzer (CTL, Shaker Heights, OH).

### Microneutralization assay

The microneutralization assay was adapted from the “WHO Serological diagnosis of influenza by neutralization assay,” as previously described.^6^ The Reed-Muench method was applied to calculate the tissue culture infectious dose (TCID_50_) for each virus strain, with or without the addition of trypsin. Briefly, equal amounts of serum from individual mice were pooled according to vaccine group and incubated at 37°C for 18–20 h with receptor-destroying enzyme (1:3) (cholera filtrate, C8872, Sigma-Aldrich). The enzyme was deactivated at 56°C for 30 min, and serial serum dilutions were added in triplicate to 96-well plates (Costar 3590, Corning, NY, USA). Virus was diluted according to the calculated TCID^42^ for each strain and added to each well. Trypsin (T1426-100MG, 2 μg/mL, Sigma-Aldrich) for the A/turkey/Italy/3889/1999 (H7N1) virus, while PR8 (H1N1) and NIBGR-14 (H5N1) assays were performed without trypsin. Back titration, virus controls, and cell controls were also included. Plates were incubated at 37°C for 2 h prior to the addition of 20 000 MDCK cells per well, followed by incubation at 37°C in 5% CO_2_ for 18–20 h. Next, cells were fixed with 80% acetone and left to air dry before washing with 0.3% Tween washing buffer. Biotinylated anti-influenza A nucleoprotein Ab was added (H16-L10-4R5, 1:1000, ATCC, Manassas, VA), and plates were developed and read as described for serum ELISA above.

### Serum transfer

BALB/c mice were vaccinated twice (weeks 0 and 5). Five weeks after the boost, blood was harvested by cardiac puncture. A new cohort of naive BALB/c mice was passively immunized via intravenous (i.v.) transfer of 200 μL pooled serum from each vaccine group. A 5×LD_50_ dose of PR8 virus was administered i.n. 24 h after passive immunization.

### T cell depletion

BALB/c mice were vaccinated twice (weeks 0 and 5) and challenged i.n*.* with a 5×LD_50_ dose of PR8 (H1N1) virus 5 weeks after the boost. At days −2, 0, and 2 relative to the day of challenge, mice received 200 μg of depleting mAbs i.p*.*: either anti-CD4 (clone GK 1.5, 0.5 mg/mL, BioXcell) or anti-CD8 (clone YTS 169.4, 0.5 mg/mL, BioXcell). Control mice received an isotype control mAb (clone C1.18.4 or LTF2, 0.5 mg/mL, BioXcell). Post-termination, spleens from indicator mice in the depleted and isotype control groups were harvested to assess T cell depletion. Splenic single-cell suspensions were stained with APC-conjugated anti-CD19 (1D3, TONBO Biosciences) (dump gate), VF450-conjugated anti-CD3 (17A2; TONBO Biosciences), phycoerythrin (PE)-conjugated anti-CD8a (2.43, TONBO Biosciences), and FITC-conjugated anti-CD4 (GK1.5, TONBO Biosciences). Stained samples were fixed in 4% paraformaldehyde and run the following day on an Attune NxT flow cytometer. Data were analyzed with FlowJo software (v7.6) (TreeStar, Ashland, OR).

### NK cell activation assay

The NK cell activation assay was performed as previously described.^14^ Briefly, ELISA 96-well plates (Costar 3590) were coated with PR8 HA protein (0.5 μg/mL, Sino Biological) at 4°C ON and blocked with sterile blocking buffer (1% BSA in PBS). Pooled serum from vaccinated mice was inactivated at 56°C for 30 min before being diluted 1:5 in sterile assay buffer (0.1% BSA, 0.005% Tween 20 in PBS), added to each well in technical triplicates, and incubated at 4°C ON. Meanwhile, NK cells were enriched from mouse spleens using an NK cell isolation Kit (Miltenyi Biotec) and activated ON in the presence of 10 ng/mL mouse IL-15. The next day, unbound serum immunoglobulins were washed away, and 0.5 × 10^5^ pre-activated NK cells were added to each well in cell media consisting of RPMI +10% fetal bovine serum (FBS), 1X brefeldin A (Thermo Fisher Scientific), 1X GolgiStop (BD Pharmingen), 2 μg/mL anti-CD107a PE (Biolegend), and 10 ng/μL IL15. Cells were incubated at 37°C for 5 h before being transferred to a 96-well V-bottom plate. Cells were then stained with GhostDyeV510 (1:400, Tonbo biosciences), anti-Nkp46 eFluor450 (2 μg/mL, Thermo Fisher Scientific), and anti-CD3e FITC (2 μg/mL, Biolegend). Lastly, cells were permeabilized and stained with anti-IFNγ APC (2 μg/mL, Tonbo Bioscience). Activated CD3e^−^Nkp46^+^ CD107a^+^IFNg^+^ NK cells were acquired by flow cytometry on an Attune NxT flow cytometer (Thermo Fisher Scientific), and data were analyzed with FlowJo software (v7.6).

### Statistical methods

Statistical calculations on endpoint titers in serum ELISA, SFC in ELISpot, and H1-reactive GC cells in flow cytometry were performed using the non-parametric Kruskal-Wallis test and Dunn’s multiple comparisons test. Comparisons of groups of pooled sera in the NK cell activation assays were analyzed using Brown-Forsyth and Welch one-way ANOVA with Dunnett’s T3 multiple comparisons test. Correlations were assessed using the non-parametric Spearman correlation. Kaplan-Meier curves were analyzed using the Gehan-Breslow-Wilcoxon test. Weight curve analyses were done using two-way ANOVA with Dunnet’s multiple comparison post-test, and significance was reported from day 8 after challenge. When mice were euthanized upon reaching the 20% weight loss limit, the last recorded weight was used for curve analysis. OD-curve analyses were performed using the unpaired *t* test of area under the curve (AUC) values. All calculations were two-tailed and performed using the GraphPad Prism 10.4.1 software.

## Data availability

Raw data and additional information will be available upon request from the corresponding author.

## Acknowledgments

We are thankful to Tor-Kristian Andersen, Kirankumar Katta, and Peter Hofgaard for their technical expertise and assistance. The following reagent was obtained through BEI Resources, NIAID, NIH: Influenza A Virus, A/Hong Kong/1/1968-2 Mouse-Adapted 21-2 (H3N2), NR-28634. This work was funded by grants from the European Union H2020 (grant no. 874866, to G.G. and B.B.), 10.13039/501100005416The Research Council of Norway (project 287872 to G.G. and project 300049 to B.B.), and the 10.13039/501100006095South-Eastern Norway Regional Health Authority, Norway (project 2021087 to G.G. and projects 2019127 and 2020046 to R.B.). No funder had any role in project design, data interpretation, or presentation of analyses.

## Author contributions

A.M.A., G.G., and B.B. conceived and designed the study and experiments. B.B. and R.B. developed the heterodimeric vaccine format. A.M.A., D.M.H., E.T., and G.G. performed the experiments and analyzed the results. A.M.A., G.G., and B.B. wrote the manuscript. All authors contributed feedback on the manuscript.

## Declaration of interests

B.B. is an inventor on granted patents related to C_H_3-based vaccines and the human MIP1a (LD78b) targeting unit and holds shares in Nykode Therapeutics. The TTO office of the University of Oslo and Oslo University Hospital, Inven2, has filed a patent application, no. 62/555,305, “Vaccine molecules,” for the A/B heterodimeric vaccine molecules. With R.B. and B.B. listed as inventors. Further, Inven2 has filed a patent on the vaccine mixture, no. 16645135, with A.M.A., G.G., and B.B. listed as inventors.

## Supplemental information

Document S1. Figures S1–S7 and Tables S1 and S2

Document S2. Article plus supplemental information
