# Supplementary material for: DNA vaccines targeting hemagglutinin from 18 subtypes of influenza A virus to antigen-presenting cells confer broad protection
Source: Mol Ther Nucleic Acids. 2025 Dec 26;37(1):102814. doi: 10.1016/j.omtn.2025.102814 (PMC12818058; doi:10.1016/j.omtn.2025.102814)
Supplement: Document S1. Figures S1–S7 and Tables S1 and S2 [file mmc1.pdf]

OMTN, Volume *37*

Supplemental information

DNA vaccines targeting hemagglutinin from 18 subtypes of influenza A virus to antigen-presenting cells confer broad protection

Ane Marie Anderson, Elias Tjärnhage, Daniëla Maria Hinke, Ranveig Braathen, Gunnveig Grodeland, and Bjarne Bogen

Table S1: Overview of influenza A subtypes and reagents.

	HA subtype	Influenza A strain	Motif in A/B-dimerized vaccines	Cat. No. rec. HA (Sino biological)	Live virus
Group 1	H1 (PR8)	H1N1 A/Puerto Rico/8/1934	-	11684-V08H	Yes
	H1 (Cal07)	H1N1 A/California/07/2009	B	11085-V08H	-
	H2	H2N2 A/Canada/720/2005	B	11688-V08H	-
	H5 (HK97)	H5N1 A/Hong Kong/483/97	B	11689-V08H	-
	H5 (Cam07)	H5N1 A/Cambodia/R0405050/2007	-	11710-V08H	-
	H5 (NIBRG-14)	H5N1 A/Vietnam/1194/2004 x Puerto Rico/8/1934	-	-	Yes
	H6	H6N1 A/northern shoveler /California/HKWF115/2007	B	11723-V08H	
	H8	H8N4 A/pintail duck/Alberta/114/1979	A	11722-V08H	-
	H9	H9N2 A/Hong Kong/1073/99	A	11229-V08H	-
	H11	H11N2 A/duck/Yangzhou/906/2002	A	11705-V08H	-
	H12	H12N5 A/green-winged teal/ALB/199/1991	A	11718-V08H	-
	H13	H13N8 A/black-headed gull/Netherlands/1/00	A	11721-V08H	-
	H16	H16N3 A/black-headed gull/Sweden/5/99	A	11711-V08H	-
	H17	H17N10 A/little yellow-shouldered bat/Guatemala/164/2009	B	40323-V08B	-
	H18	H18N11 A/flat-faced bat/Peru/033/2010	B	40324-V08B	-
Group 2	H3	H3N2 A/Hong Kong/1/1968	B	40116-V08B	-
	H3 (HK68-MA21)	H3N2 A/Hong Kong/1/1968-MA21			Yes
	H4	H4N6 A/Swine/Ontario/01911-1/99	B	11706-V08H	-
	H7 (ITA99)	H7N1 A/chicken/Italy/13474/1999	-	-	-
	H7 (SH1)	H7N9 A/Shanghai/1/2013	B	40104-V08H	-
	H7 (ITA3889)	H7N1 A/turkey/Italy/3889/1999	-	-	Yes
	H10	H10N3 A/duck/Hunan/S11205/2012	A	40360-V08H	-
	H14	H14N5 A/Mallard/Astrakhan/263/1982	A	40192-V08B	-
	H15	H15N8 A/duck/AUS/341/1983	A	11720-V08H	-

Table S2: Alignments of H1, H5 and H7 subtypes. HA sequence alignments of (**A**) H1 from subtypes PR8 and Cal07, (**B**) H7 from subtypes ITA99, ITA3889, and SH1, and (**C**) H5 from subtypes HK97, NIRBG-14, and Cam07. Alignments were made using the blastp suite from https://blast.ncbi.nlm.nih.gov/Blast.cgi. aa numbering follows query sequence, e.g. PR8 in **A**, HK97 in **B**, and H7 ITA99 in **C**. The conserved HA stem fusion peptide is marked in grey. ΔRVRR denotes the location of the multibasic cleavage site (MBS) that was removed from the H7 (ITA99) vaccine HA sequence.

A

H1

```
PR8        18   DTICIGYHANNSTDTVDTVLEKNVTVTHSVNLLEDSHNGKLCRLKGIAPLQLGKCNIAGW  77
Cal07           ..L................................K......K.R.V...H.........

PR8        78   LLGNPECDPLLPVRSWSYIVETPNSENGICYPGDFIDYEELREQLSSVSSFERFEIFPKE  137
Cal07           I......ES.STAS.........S.D..T..............................T

PR8        138  SSWPNHNTTKGVTAACSHAGKSSFYRNLLWLTEKEGSYPKLKNSYVNKKGKEVLVLWGIH  197
Cal07           ......DSN.......P...AK...K..I..VK.GN.....SK..I.D............

PR8        198  HPSNSKDQQNIYQNENAYVSVVTSNYNRRFTPEIAERPKVRDQAGRMNYYWTLLKPGDTI  257
Cal07           ...T.A...SL...AD...F.GS.R.SKK.K....I......RE.........VE...K.

PR8        258  IFEANGNLIAPRYAFALSRGFGSGIITSNASMHECNTKCQTPLGAINSSLPFQNIHPVTI  317
Cal07           T...T...VV......ME.NA.....I.DTPV.D...T....K....T.........I..
                                       HA1\/HA2
PR8        318  GECPKYVRSAKLRMVTGLRNIPSIQSRGLFGAIAGFIEGGWTGMIDGWYGYHHQNEQGSG  377
Cal07           .K.....K.T...LA.............................V...............

PR8        378  YAADQKSTQNAINGITNKVNSVIEKMNIQFTAVGKEFNKLEKRMENLNKKVDDGFLDIWT  437
Cal07           ....L.......DE.............T..........H....I................

PR8        438  YNAELLVLLENERTLDFHDSNVKNLYEKVKSQLKNNAKEIGNGCFEFYHKCDNECMESVR  497
Cal07           ................Y............R.......................T.....K

PR8        498  NGTYDYPKYSEESKLNREKVDGVKLESMGIYQILAIYSTVASSL  541
Cal07           ............A.....EI.......TR...............
```

B

H7

```
ITA99      19   DKICLGHHAVSNGTKVNTLTERGVEVVNATETVERTNVPRICSKGKRTVDLGQCGLLGTI  78
ITA3889         ............................................................
SH1             .....................................I......................

ITA99      79   TGPPQCDQFLEFSADLIIERREGSDVCYPGKFVNEEALRQILRESGGIDKEAMGFTYSGI  138
ITA3889         ........................G..........................T........
SH1             ............................................................

ITA99      139  RTNGTTSTCRRSGSSFYAEMKWLLSNTDNAAFPQMTKSYKNTRKDPALIIWGIHHSGSTT  198
ITA3889         .......A...L................................................
SH1             ....A..S....................................N....V.........A

ITA99      199  EQTKLYGSGNKLITVGSSNYQQSFVPSPGERPQVNGQSGRIDFHWLMLNPNDTVTFSFNG  258
ITA3889         .............................A..............................
SH1             ............V................A.T....L.......................

ITA99      259  AFIAPDRASFLRGKSMGIQSGVQVDANCEGDCYHSGGTIISNLPFQNINSRAVGKCPRYV  318
ITA3889         ............................................................
SH1             ..........................D......Y..............D...........
                                     ΔRVRR (MBS)
                                 HA1\/HA2
ITA99      319  KQESLLLATGMKNVPEIPKGSGLFGAIAGFIENGWEGLIDGWYGFRHQNAQGEGTAADYK  382
```

```
ITA3889                ................V...R.......................................
SH1                    ..R.............Q...R.......................................

ITA99       383        STQSAIDQVTGKLNRLIEKTNQQFELIDNEFTEVEKQIGNVINWTRDSMTEVWSYNAELL   442
ITA3889                ............................................................
SH1                    ........I.......................................I...........

ITA99       443        VAMENQHTIDLTDSEMNKLYERVKRLLRENAEEDGTGCFEIFHKCDDDCMASIRNNTYDH   502
ITA3889                ...........A.............Q..................................
SH1                    ...........A....D........Q..................................

ITA99       503        SKYREEAMQNRIQIDPVKLSSGYKDVILWFSFGA   536
ITA3889                ..................................
SH1                    ..................................
```

C

H5

```
HK97        17         DQICIGYHANNSTEQVDTIMEKNVTVTHAQDILERTHNGKLCDLNGVKPLILRDCSVAGW   76
NIBRG-14               ..................................K.........D...............
Cam07                  ..................................K.........D...............

HK97        77         LLGNPMCDEFINVPEWSYIVEKASPANDLCYPGNFNDYEELKHLLSRISHFEKIQIIPKS   136
NIBRG-14               .......................N.V.......D..............N...........
Cam07                  .......................N.V.......D..............N...........

HK97        137        SWSNHDASSGVSSACPYLGKSSFFRNVVWLIKKNSTYPTIKRSYNNTNQEDLLVLWGIHH   196
NIBRG-14               ...S.E..L........Q..........................................
Cam07                  ..PS.E..L...A....Q................N...................M.....

HK97        197        PNDAAEQTKLYQNPTTYISVGTSTLNQRLVPEIATRPKVNGQSGRIEFFWTILKPNDAIN   256
NIBRG-14               ...............................R....S........M..............
Cam07                  ........N....................T.R....S........M..............

HK97        257        FESNGNFIAPEYAYKIVKKGDSTIMKSELEYGNCNTKCQTPMGAINSSMPFHNIHPLTIG   316
NIBRG-14               ............................................................
Cam07                  ............................................................
                                                 HA1\/HA2
HK97        317        ECPKYVKSNRLVLATGLRNAPQRERRRKKRGLFGAIAGFIEGGWQGMVDGWYGYHHSNEQ   376
NIBRG-14               ...................S........................................
Cam07                  ...................S....G...................................

HK97        377        GSGYAADQESTQKAIDGVTNKVNSIINKMNTQFEAVGREFNNLERRIENLNKKMEDGFLD   436
NIBRG-14               .......K..................D.................................
Cam07                  .......K..................D.................................

HK97        437        VWTYNAELLVLMENERTLDFHDSNVKNLYDKVRLQLRDNAKELGNGCFEFYHKCDNECME   496
NIBRG-14               ............................................................
Cam07                  ............................................................

HK97        497        SVKNGTYDYPQYSEEARLNREEISGVKLESMGTYQILSLYSTVASSL   543
NIBRG-14               ..R...............K...........I.I.....I........
Cam07                  ..R...............K...........I.I.....I........
```

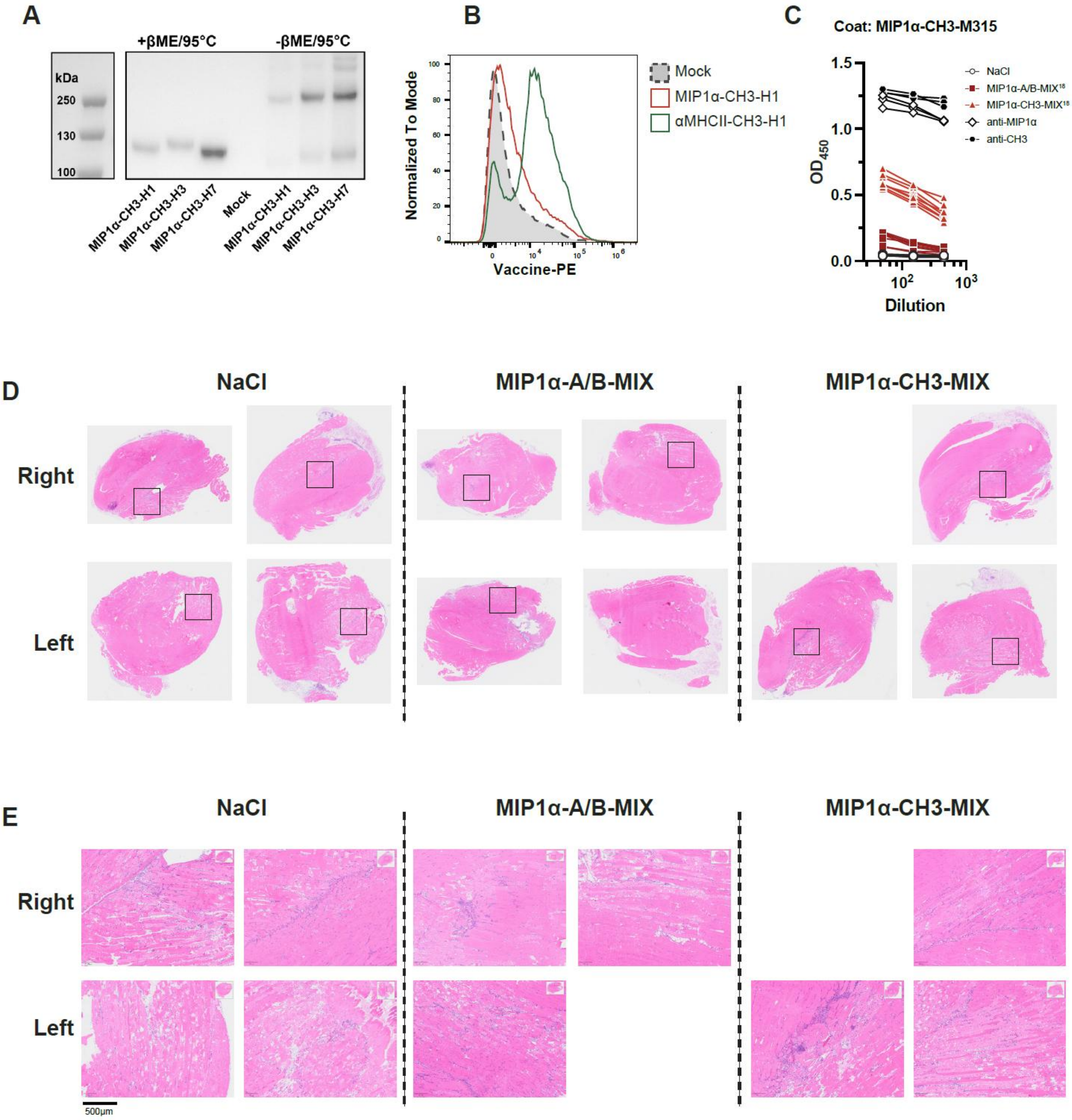

Figure S1: Vaccine proteins, and evaluation of vaccination sites. (**A**) Western Blot of vaccine proteins in supernatants of transiently transfected HEK293E cells were performed under standard denaturing conditions (left) or non-denaturing conditions (no addition of b-ME and boiling, right). (**B**) CD11b^{+} splenocytes from BALB/c mice were stained with supernatants from HEK293E cells transiently transfected with plasmids encoding the indicated vaccine proteins. Vaccine bound cells were detected with secondary anti-HA staining, and evaluated by flow cytometry. (**C**) Mice were vaccinated twice with MIP1α-C_H3-MIX[18], MIP1α-A/B-MIX[18], or saline, and sera collected at day 28 evaluated for reactivity against the vaccine backbone of MIP1α and the C_H3 based dimerization unit (coat: vaccine proteins equipped with MIP1α as targeting unit, the C_H3 based dimerization unit, and an irrelevant antigen, M315). Antibodies against the C_H3 domain and MIP1α was used as positive controls. (**D**) Muscle fibers from the site of vaccination were collected 24 hours post vaccination, and stained for Hematoxylin and Eosin (H&E) to examine tissue inflammation and potential damage. Highlighted squares indicate the areas of most inflammation, and that has been magnified in (**E**).

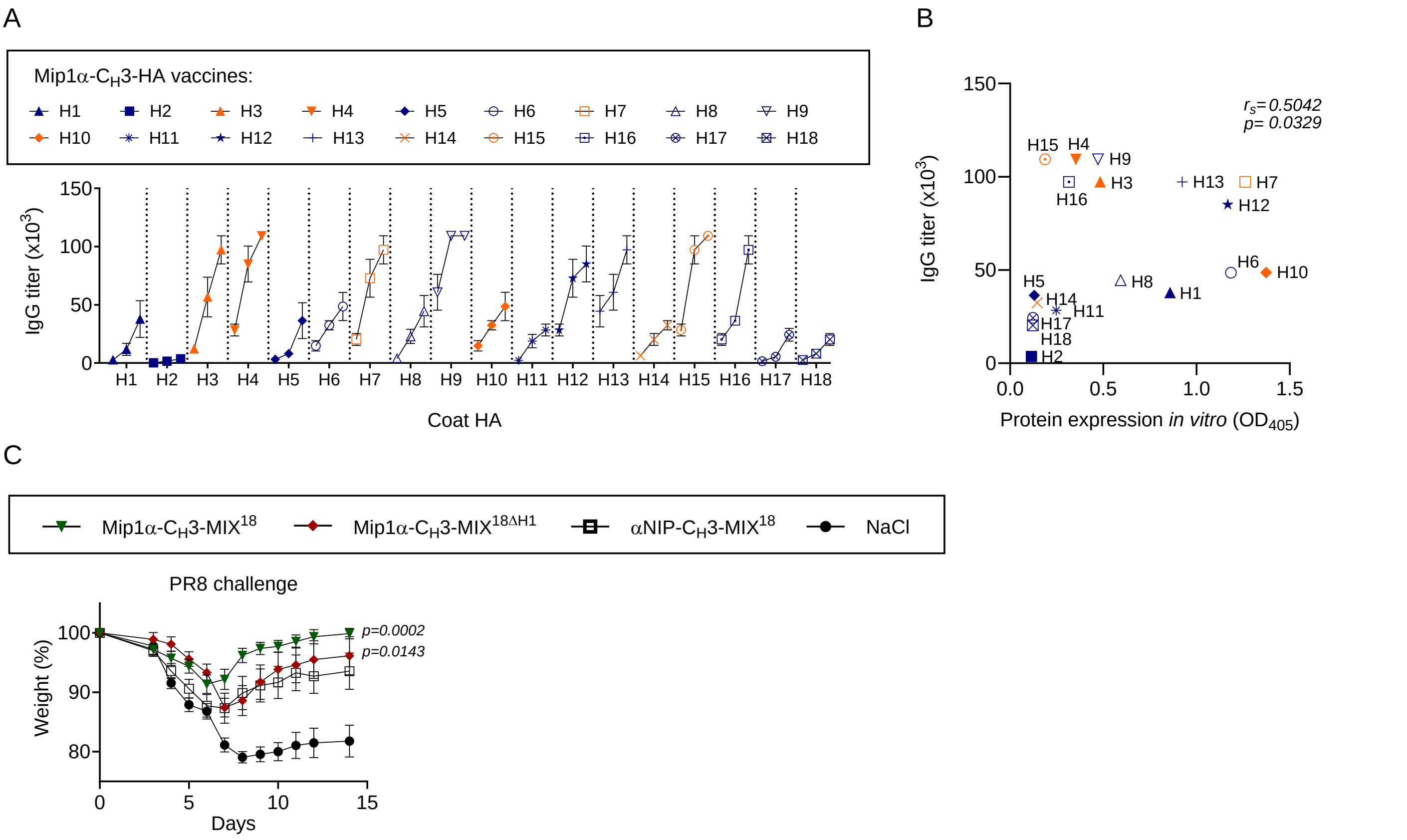

Figure S2: *In vivo* data from mice. (**A**) BALB/c mice (n=6/group) were vaccinated once with 20 µg DNA encoding one of the Mip1α-C$_H$3-HA vaccines, each expressing a selected HA from influenza H1-H18 (influenza group 1 in blue, group 2 in orange). Sera from individual mice were analyzed for IgG against homologous HA (heterologous SH1 for H7) at 2, 4 and 6 weeks after vaccination (mean ± SEM). (**B**) HEK293E cells were transiently transfected with 1µg from each of the indicated Mip1α-C$_H$3-HA plasmids, and secreted vaccine proteins were detected by ELISA (mean ± SEM of technical triplicates) and correlated to the *in vivo* IgG responses from (A) at 6 weeks after vaccination (mean ± SEM). (**C**) BALB/c mice (n= 10/group) were vaccinated twice at weeks 0 and 5 (arrows) with the indicated plasmid mixtures. Five weeks after the boost, mice were challenged with PR8 influenza virus (5xLD$_{50}$) and monitored for weight loss.

Correlation in (B) was performed using a two-way Spearman correlation. Statistical significance in (C) was calculated with a two-way Anova with Dunnett's multiple comparisons test for each group compared to NaCl controls, and the values shown are for day 8.

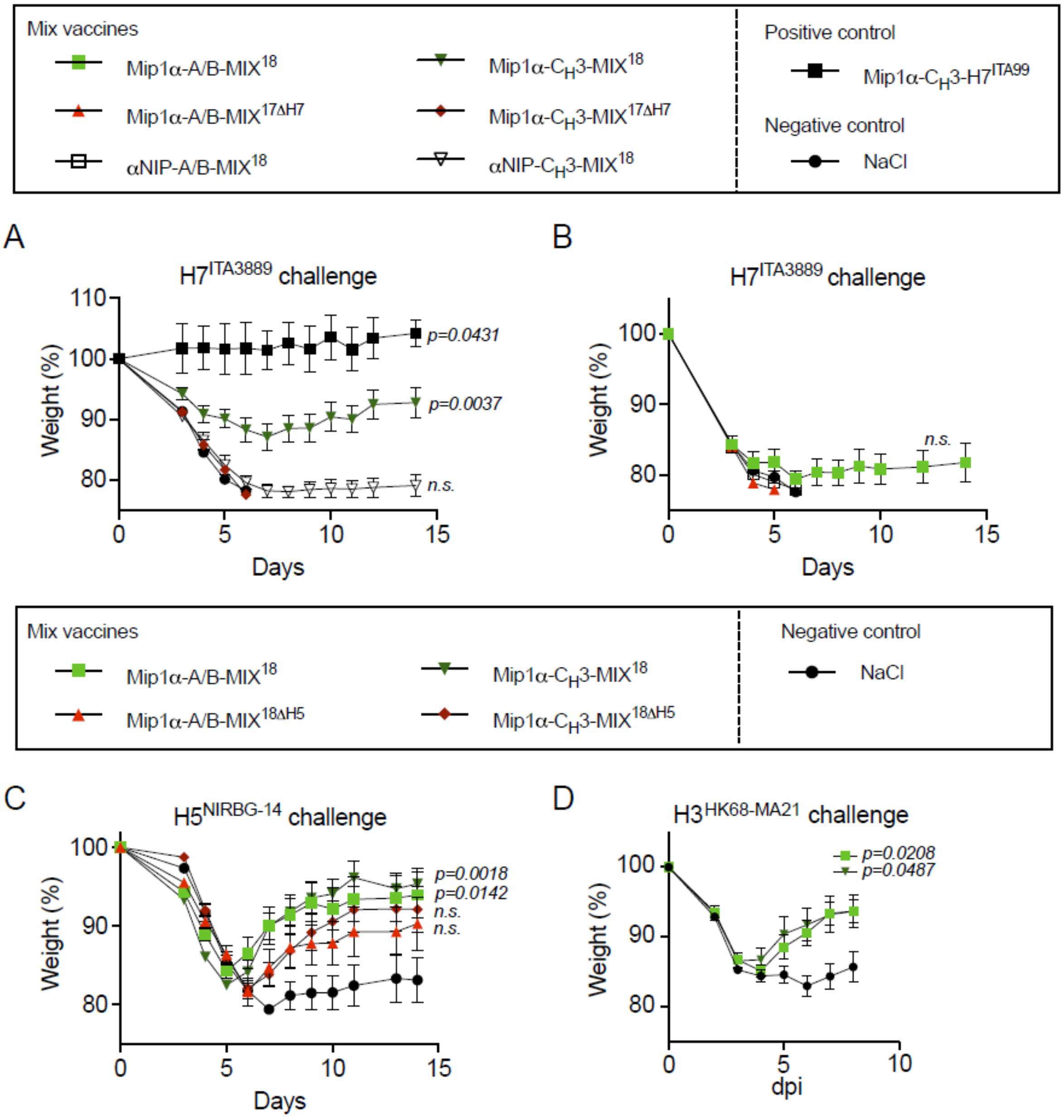

Figure S3: MIP1α-targeted plasmid mixtures cross-protect against heterologous H7 and H5 influenza viruses, and homologous H3N2. (**A-B**) In two separate experiments, BALB/c mice (n=10/group, n=4 for the Mip1α-C$_H$3-H7^{ITA99} positive control) were vaccinated twice at weeks 0 and 5 with the indicated vaccines. Five weeks after the boost, the mice were challenged with a heterologous A/turkey/Italy/3889/1999 (H7N1) influenza virus (5xLD$_{50}$) and monitored for weight loss. (**C**) BALB/c mice (n=10/group) were vaccinated twice at weeks 0 and 4 with the indicated vaccines. Five weeks after the boost, mice were challenged with a heterologous NIBRG-14 (H5N1) influenza virus (5xLD$_{50}$) and monitored for weight loss. (**D**) BALB/c mice (n=8/group) were vaccinated twice at weeks 0 and 4 with the indicated vaccines. Five weeks after the boost, mice were challenged with a homologous H3N2 influenza virus (1xLD$_{50}$) and monitored for weight loss. (**C** and **D**) Statistical significance was calculated with a two-way Anova with Dunn's multiple comparisons test for each group compared to NaCl controls, values are shown for day 8.

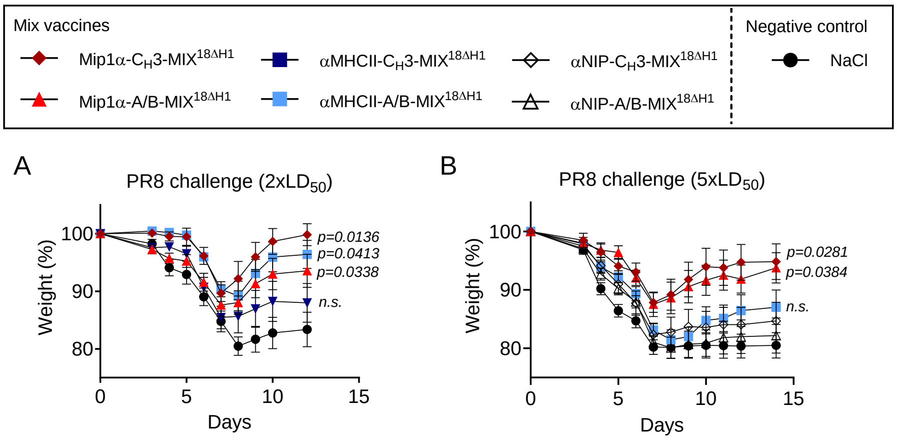

Figure S4: **Mip1α-targeting improves heterosubtypic cross-protection against H1 virus.** BALB/c mice were vaccinated twice at weeks 0 and 5 with the indicated plasmid mixtures. Five weeks after the boost, mice were challenge with PR8 influenza virus at either (**A**) 2xLD$_{50}$ (n=9-10/group) or (**B**) 5xLD$_{50}$ (n=9-10/group) and monitored for weight loss.

Statistical significance was calculated with a two-way Anova with Dunnetts's multiple comparisons test for each group compared to NaCl controls, values are shown for day 8.

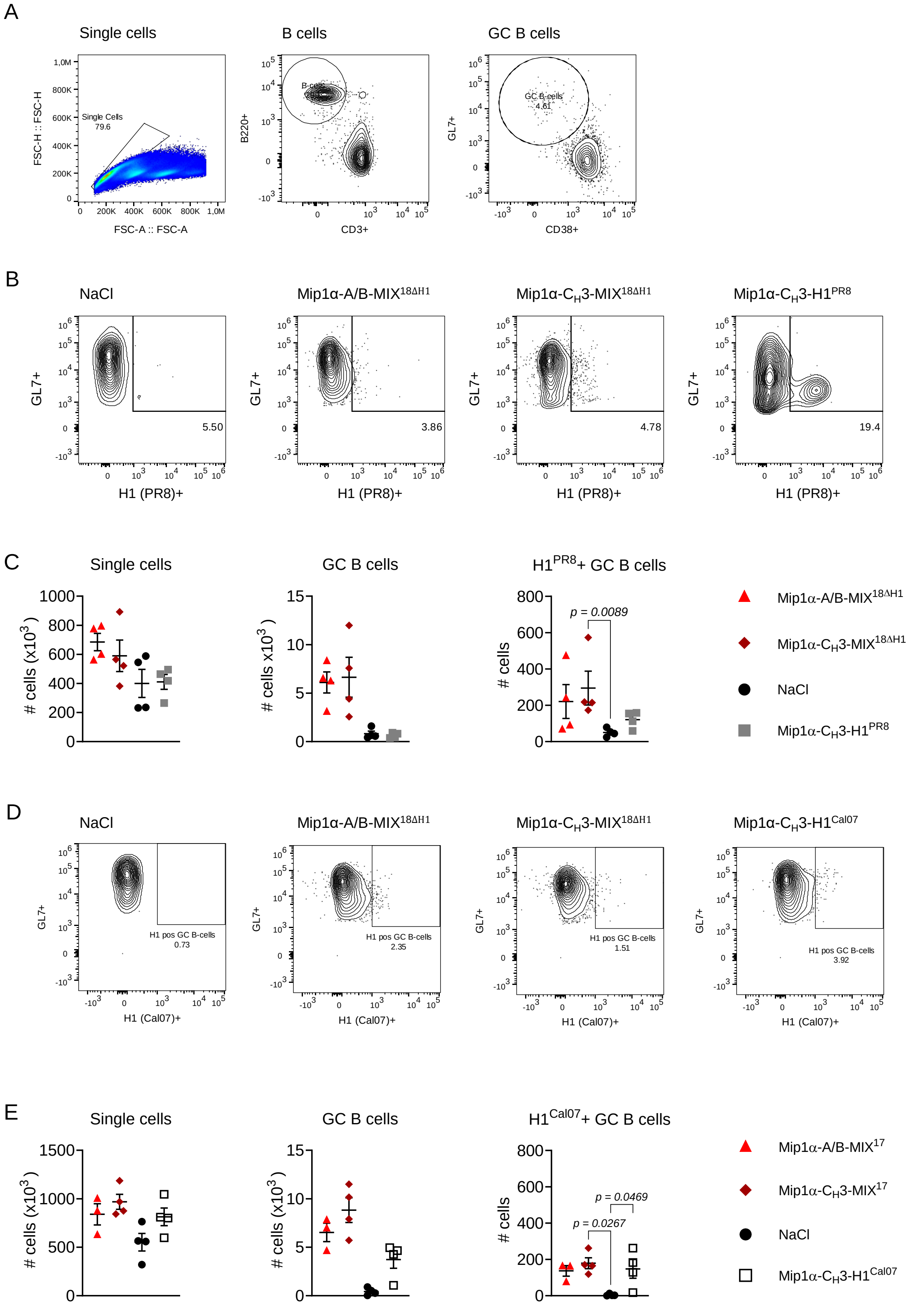

Figure S5: Gating strategy and populations for H1-reactive GC B-cells. BALB/c mice were vaccinated twice at weeks 0 and 5 with the indicated vaccines (n=4/group, n=3 for Mip1α-A/B-MIX$^{18\Delta H1}$ in E). (**A**) Gating strategy for GC B-cells (CD3$^-$B220$^+$CD38$^-$GL7$^+$). (**B**) Representative panels of H1^{PR8} reactive GC B cells for the indicated vaccine groups. (**C**) Frequencies of the cell populations used to calculate fractions in fig. 5A, PR8. (**D**) Representative panels of H1^{Cal07} reactive GC B cells for the indicated vaccine groups. (**E**) Frequencies of the cell populations used to calculate fractions in fig. 5A, Cal07. Statistical significance in (C) and (E) was calculated using the Kruskal-Wallis and Dunn's multiple comparisons test.

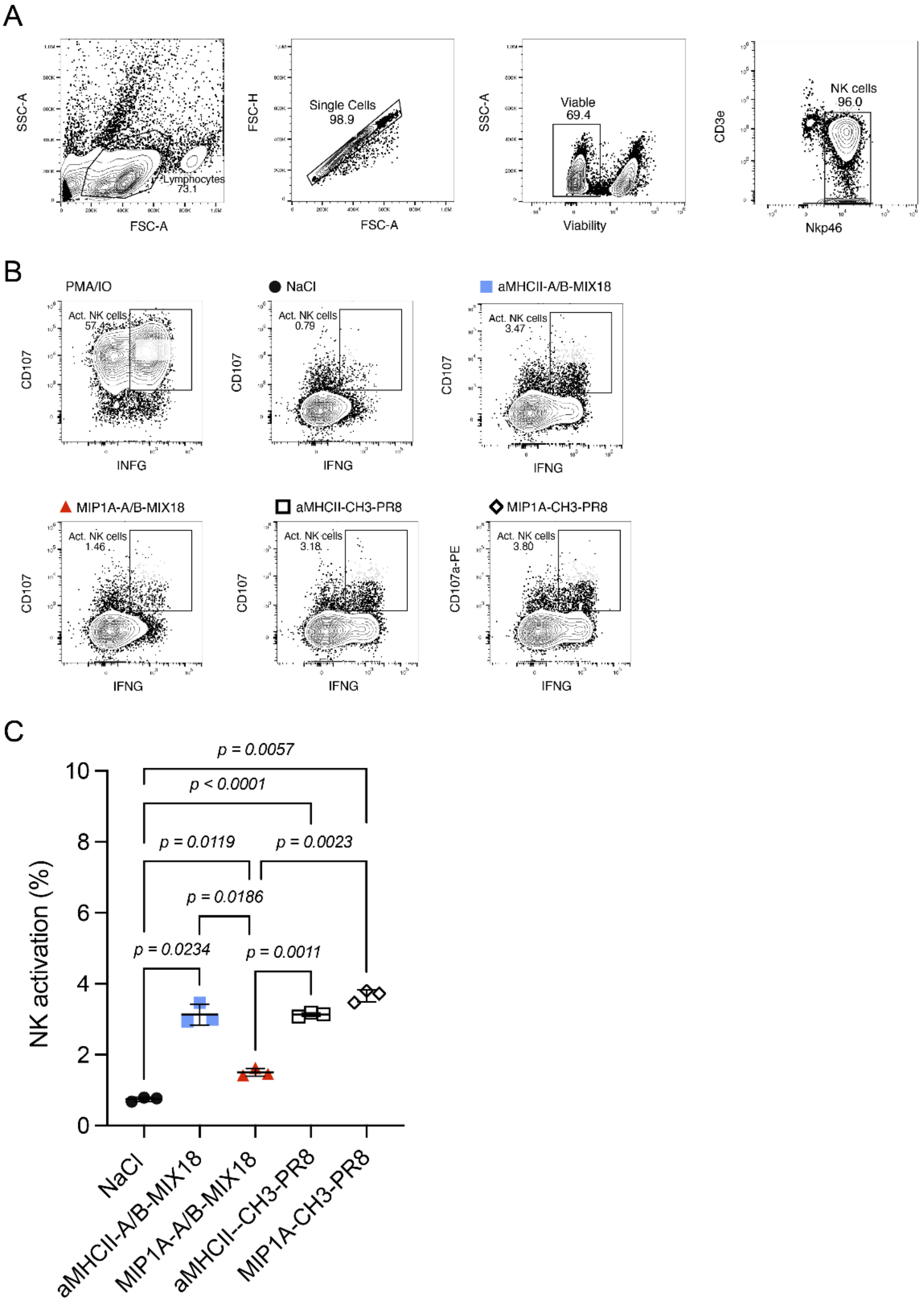

Figure S6: Antibody dependent cellular cytotoxicity by NK cell activation. Serum from vaccinated mice (n=10/group) were pooled and tested for NK cell activation *in vitro* (in triplicates). Briefly, serum immunoglobulins were bound by PR8-coated microtiter plates, unbound immunoglobulins were washed away, and NK cells isolated from BALB/c spleens were added. NK cell activation was determined by staining for activation markers and analyzed by flow cytometry. (**A**) Gating strategy for NK cells. Viable single cell lymphocytes were gated for Nkp46$^+$, CD3e$^-$. (**B**) Representative plots for activated NK cells, as determined by IFNγ$^+$, CD107a$^+$ staining. (**C**) Summary of NK activation from B. Statistical significance was determined by Brown-Forsythe and Welch 1-way ANOVA, with Dunnett's T3 multiple comparison tests.

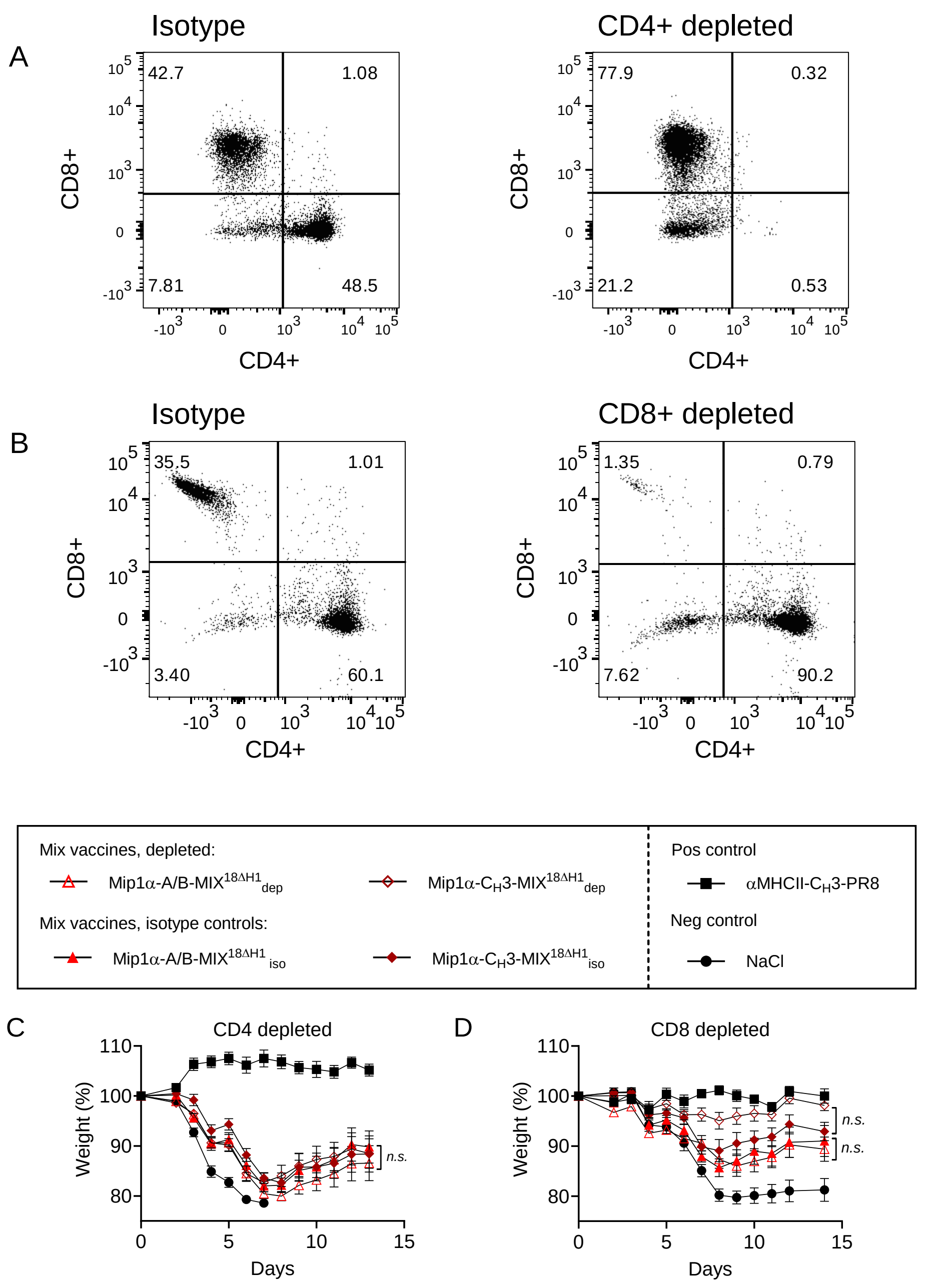

Figure S7: Depletion of T cells. In two separate experiments, BALB/c mice (n=10/group) were vaccinated twice at weeks 0 and 5 with the indicated vaccines. Five weeks after the second vaccination, mice were challenged with a 5xLD_{50} dose of PR8 influenza and monitored for weight loss (mean ± SEM). At days -2, 0 and +2 relative to challenge, mice were injected with either anti-CD4 or anti-CD8 depleting mAb, or an isotype matched control. Depletion of (**A**) $CD4^+$ or (**B**) $CD8^+$ T cells was confirmed by analysis of splenic cell suspensions from indicator mice in flow cytometry. (**C-D**) Following viral influenza challenge, mice were monitored for weight loss.

Statistical significance was calculated with a two-way Anova with Dunnett’s multiple comparisons test for each group compared to NaCl controls, values are shown for day 8.
